# Supplementary material for: Chronic macrophage activation derails muscle repair by disrupting mannose-receptor-linked plasticity revealed by endogenous irg1/acod1 tracking
Source: Nat Commun. 2026 Jan 7;17:1466. doi: 10.1038/s41467-025-68204-3 (PMC12886890; doi:10.1038/s41467-025-68204-3)
Supplement: Supplementary file 1 — Supplementary Information [file 41467_2025_68204_MOESM1_ESM.pdf]

Supplemental Information for

Chronic macrophage activation derails muscle repair by disrupting mannose-receptor-linked plasticity revealed by endogenous *irg1/acod1* tracking

Caroline G. Spencer[‡1], Matthew Hamilton[‡1], Ethan Bedsole[1], Yingshan N. Wei[1], Alison M. Rojas[1], Andrew Burciu[1], John Zhu[1], Keith Z. Sabin[1] and Celia E. Shiau[1, 2]*

[1]Department of Biology, University of North Carolina at Chapel Hill, Chapel Hill, NC
[2]Department of Microbiology and Immunology, University of North Carolina at Chapel Hill, Chapel Hill, NC

[‡] These authors contributed equally.
* Corresponding author
Email: shiauce@unc.edu

This file contains:
Supplementary Figures 1-21
Supplementary References for this file

Supplementary Figure 1

a

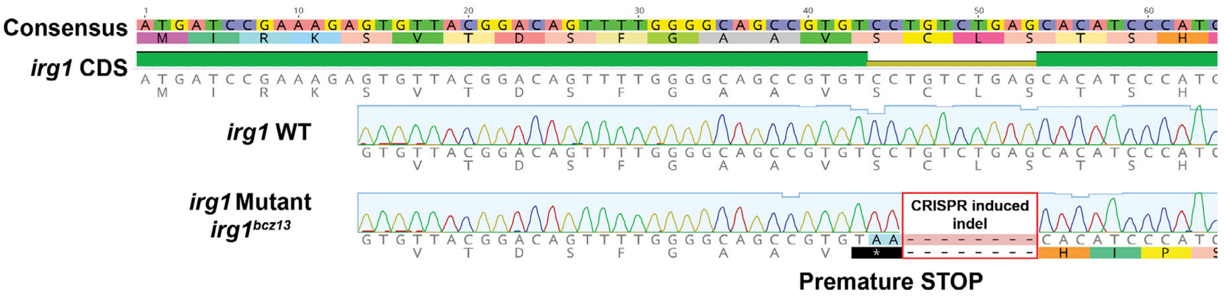

b

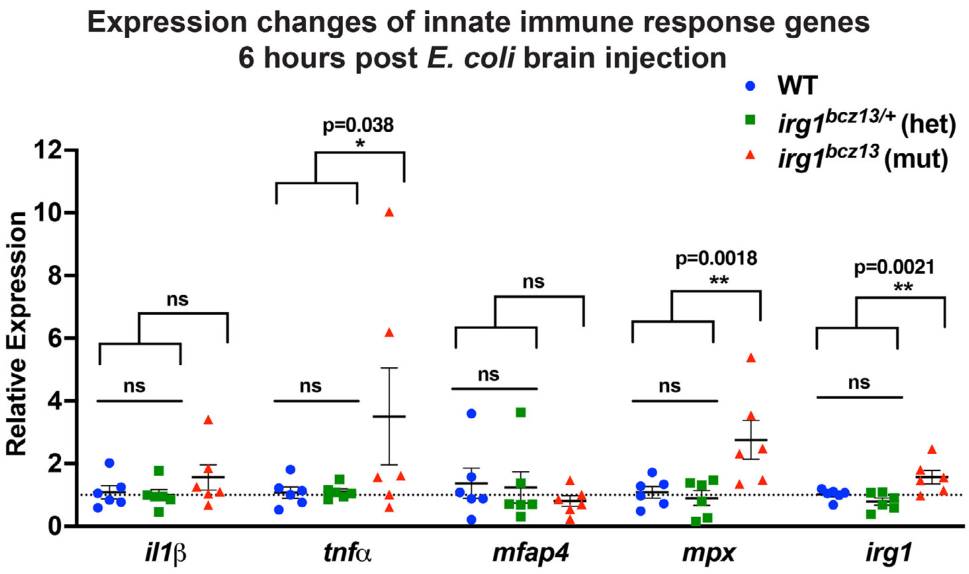

c

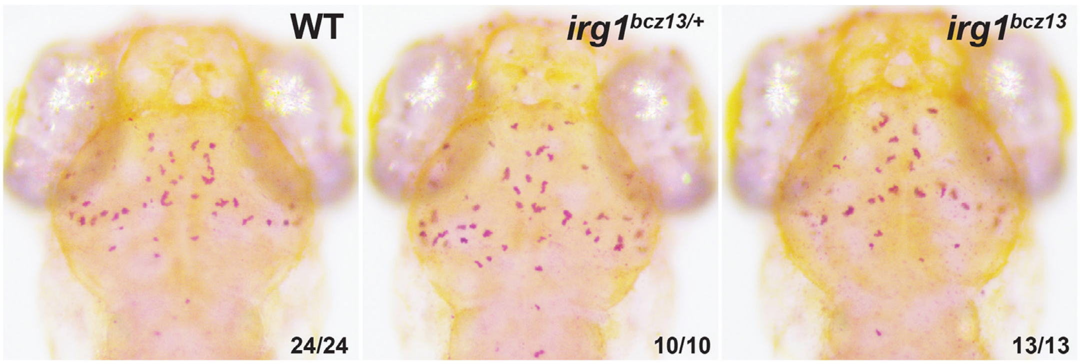

Supplementary Figure 1. Nonsense mutation *bcz13* in zebrafish *irg1/acod1* created by CRISPR-Cas9 confirms equivalent heterozygous and wild-type phenotypes.

a Sanger sequencing of *bcz13* mutation shows an indel causing a premature stop codon near the start of the *irg1* open reading frame. **b** Immune response at 6 hours post LPS injection shows comparable gene expressions between heterozygotes and wild types in contrast to the significantly altered expression levels in homozygous *bcz13* mutants. Sample size is *n* = 6 animals per genotype. Data shows mean +/- SEM. Statistical significance was determined by two-tailed student's t-test comparing between mutants and siblings (wild-type and heterozygotes combined). **c** Assessment by neutral red vital dye staining shows normal microglia development in all genotypes. Number of animals with wild-type microglial pattern out of total *n*, number of animals analyzed, is indicated in the lower right corner. ns, not significant.

Supplementary Figure 2

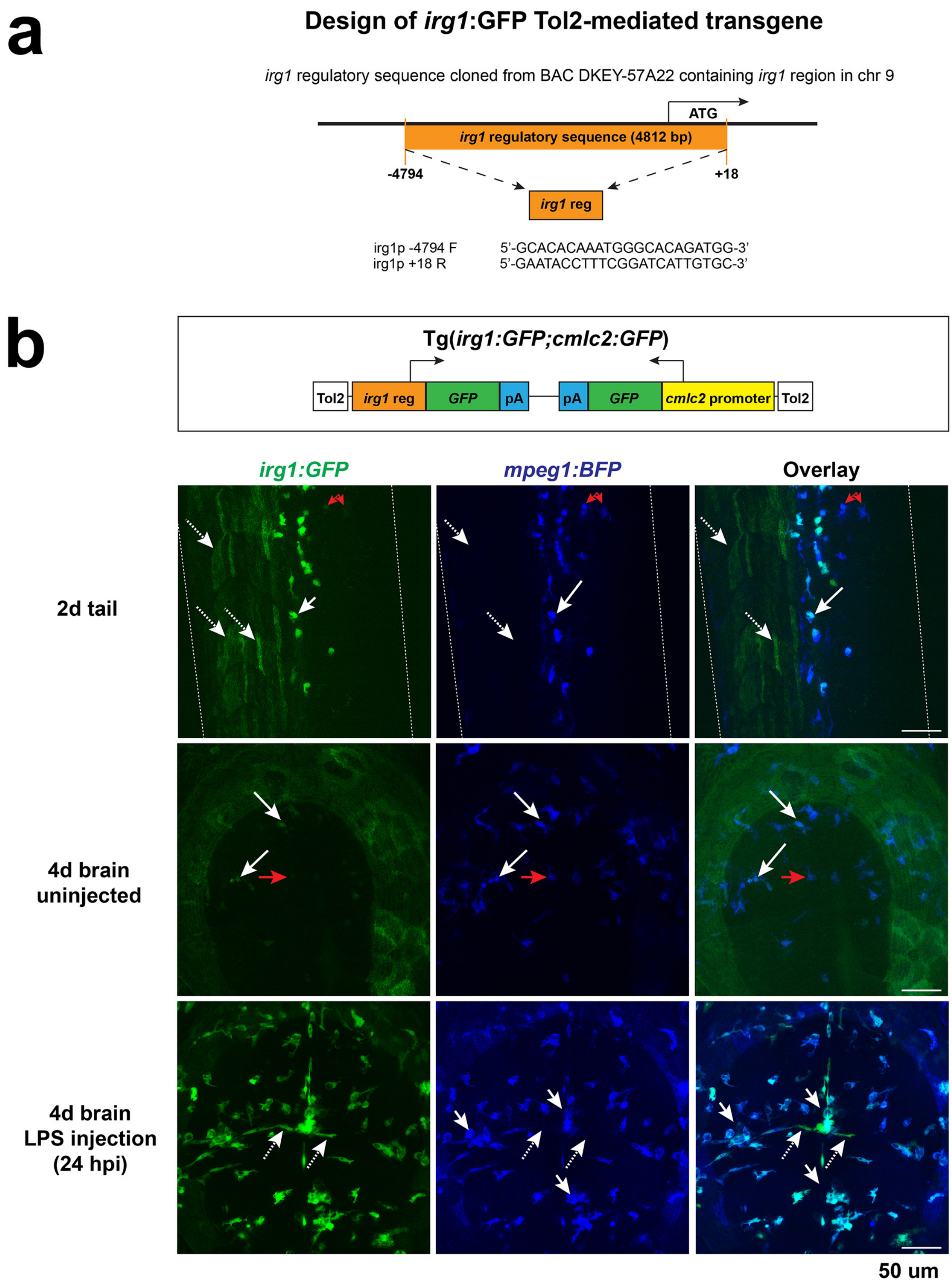

Supplementary Figure 2. Tol2-mediated exogenous *irg1:GFP* reporter shows GFP induction in activated macrophages in the brain and periphery after LPS but also off-target expression.

a Tol2-mediated transgenesis of an *irg1:GFP* reporter was made using a 4.8 kb regulatory sequence of *irg1* cloned from a BAC plasmid. **b** Tol2 reporter line was made using a plasmid with flanking tol2 transposable elements and two expression cassettes: *irg1:GFP* and *cmlc2:GFP* oriented in opposite directions, the latter provides a heterologous marker to sort transgenic fish based on a heart GFP expression. GFP expression is observed to be variable, whereby baseline macrophages in the tail can have strong GFP expression (white arrow) with few expressing nearly no GFP (red arrows). LPS-injected brains can robustly induce GFP in activated microglia (white arrows), but also off-target GFP expression in non-macrophage cells such as myocytes and other cell types that normally do not express *irg1* (white arrows with dotted lines). Red arrow, macrophages with minimal to no GFP. White arrow, macrophages with strong GFP.

Supplementary Figure 3

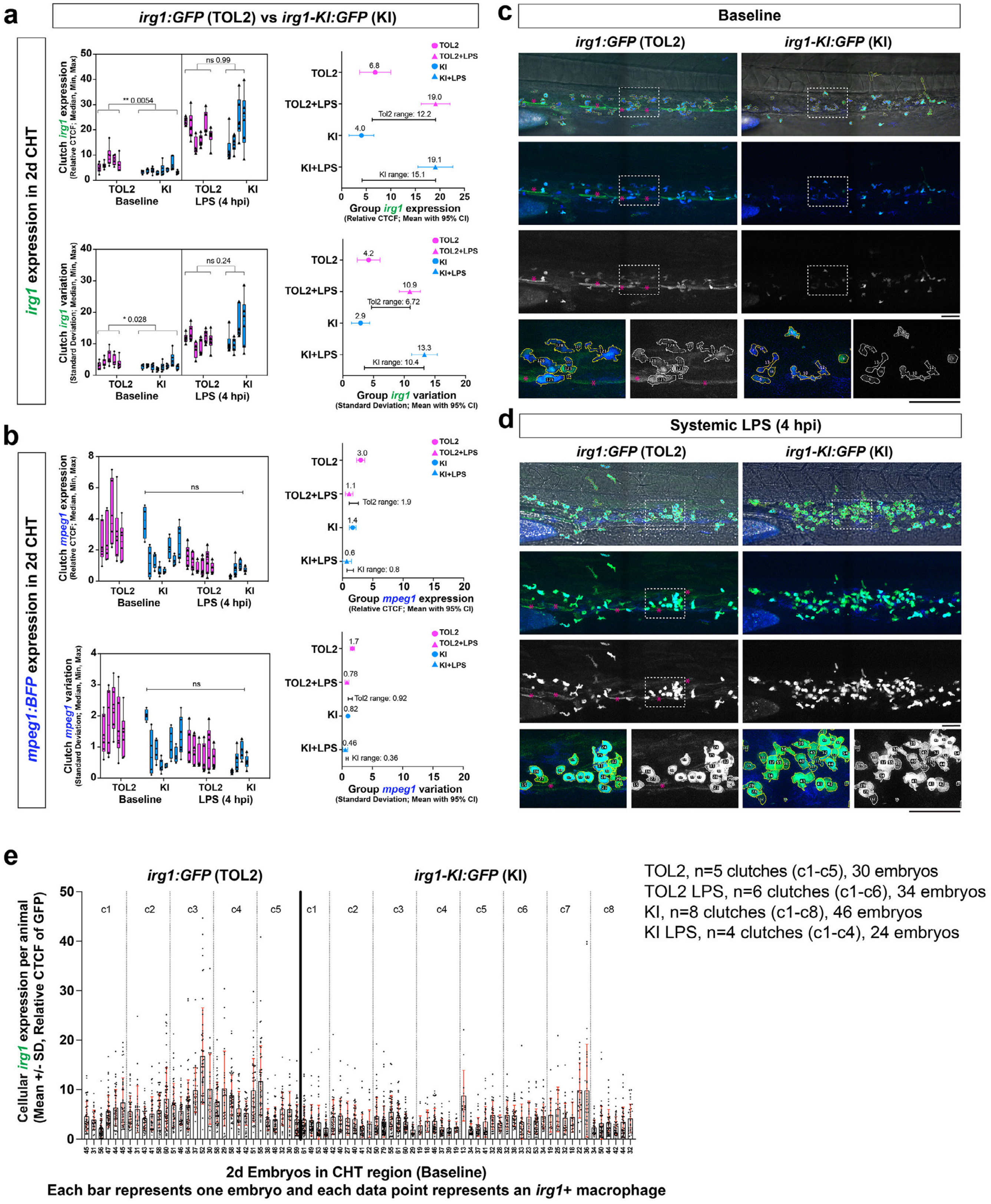

Supplementary Figure 3. GFP knock-in (KI) construct provides a more consistent and specific immune cell activation reporter than the Tol2 reporter.

a Comparison of GFP intensity and variablity (standard deviation) from the KI and Tol2 reporters provides a measure of within- and between- clutch variation at baseline and after immune activation at 4 hours post-injection (hpi) of LPS. Right, plots show standard deviation of the graphs on the left. **b** Comparison of BFP intensity and variability (standard deviation) from pan-macrophage reporter *mpeg1:BFP* serves as a negative control since it is expected to be constitutively and uniformly expressed by macrophages. Data shows no significant change in levels at baseline or after LPS injection at 4 hpi as expected. **c-d** Representative confocal images show dual labeling of macrophages comparing between KI and Tol2 reporters at baseline (**c**) or after LPS injection at 4 hpi (**d**). Macrophage cell ROIs were manually outlined in ImageJ for quantification. The dotted box indicates the region of interest shown at higher magnification in the lower panels to more clearly visualize cell ROIs and annotations. Magenta asterisks denote off-target GFP expression in non-macrophage cells, frequently observed in muscle cells. **e** The scatter bar chart displays GFP fluorescence levels (relative CTCF) for all macrophages quantified per embryo, with each bar representing an individual embryo at baseline. Overall, KI embryos show consistently lower baseline GFP signal compared with TOL2 embryos. Bar graphs are grouped by clutch and condition. Numbers below bar shows n, number of cells analyzed. Relative CTCF (corrected total cell fluorescence) was calculated as the difference between the GFP integrated density (IntDen) of the target cell (cell ROI area × mean cell fluorescence) and the background IntDen (cell ROI area × mean background fluorescence), normalized to the background IntDen. All "group" plots show values for mean of each condition +/- 95% confidence interval (CI). Each clutch represents offspring from a distinct parental pair. Scale bars, 50 μm.

Supplementary Figure 4

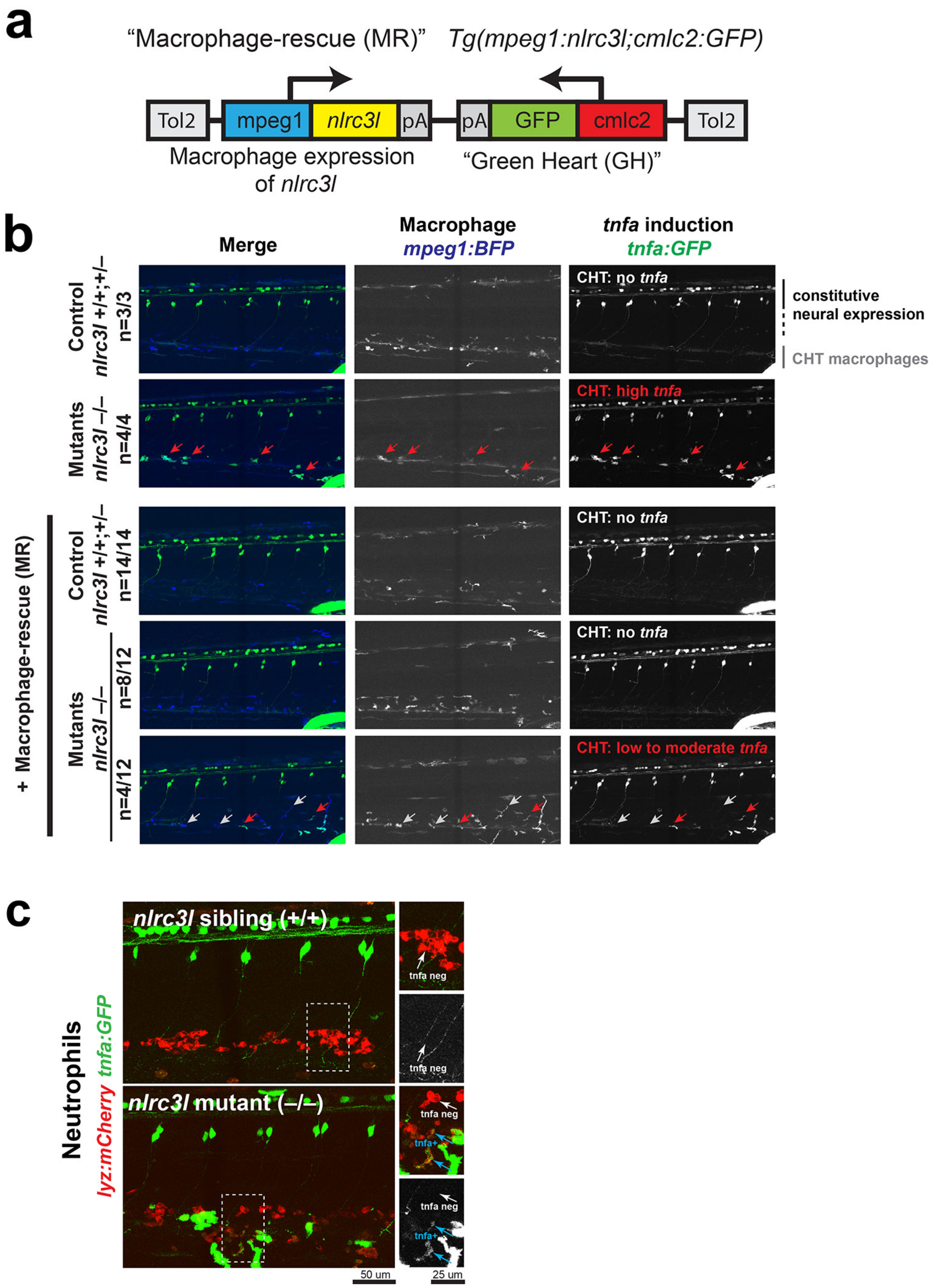

Supplementary Figure 4. Inappropriate pro-inflammatory *tnfa* induction in *nlrc3l* mutant macrophages, similar to *irg1/acod1*, is largely driven by cell-autonomous mechanisms.

a Diagram of the transgenic construct used to restore macrophage wild-type *nlrc3l* expression. Construct mediates macrophage expression of wild-type *nlrc3l* and a heterologous marker *cmlc2:GFP* that drives heart GFP expression. **b** Representative confocal maximum projection images of 3 dpf zebrafish tail showing analysis of macrophages in the caudal hematopoietic tissue (CHT) using *mpeg1:BFP* as a macrophage reporter and *tnfa:GFP* as a marker for inflammatory activation. Constitutive *tnfa:GFP* expression in the nervous system (annotated in the top row) served as a heterologous marker for confirming presence of transgene, as wild-type macrophages typically do not express *tnfa* at homeostasis. Control embryos, regardless of the presence of the macrophage-rescue (MR) cassette, showed no *tnfa* expression in macrophages. In contrast, baseline *nlrc3l* mutants exhibited high *tnfa* expression, whereas macrophage-rescued *nlrc3l* mutants mostly lacked *tnfa* expression, with approximately 33% of them showing low to moderate levels of *tnfa* in a subset of macrophages (red arrows) and remaining macrophages no *tnfa* (white arrows). This provides direct evidence that the inappropriate *tnfa* expression results from a macrophage-cell-autonomous function of *nlrc3l*. The incomplete reversal of *tnfa* induction in macrophage-rescued mutants may stem from differential MR transgene expression levels in macrophages or potential contributions of non-macrophage factors to the mutant inflammatory phenotype. **c** Double transgenic fish with the *tnfa* and neutrophil reporters show atypical expression pattern where weak *tnfa* expression is observed in select few neutrophils in *nlrc3l* mutants at baseline (blue arrow) compared with no expression in controls (white arrows). *n*, number of animals analyzed.

Supplementary Figure 5

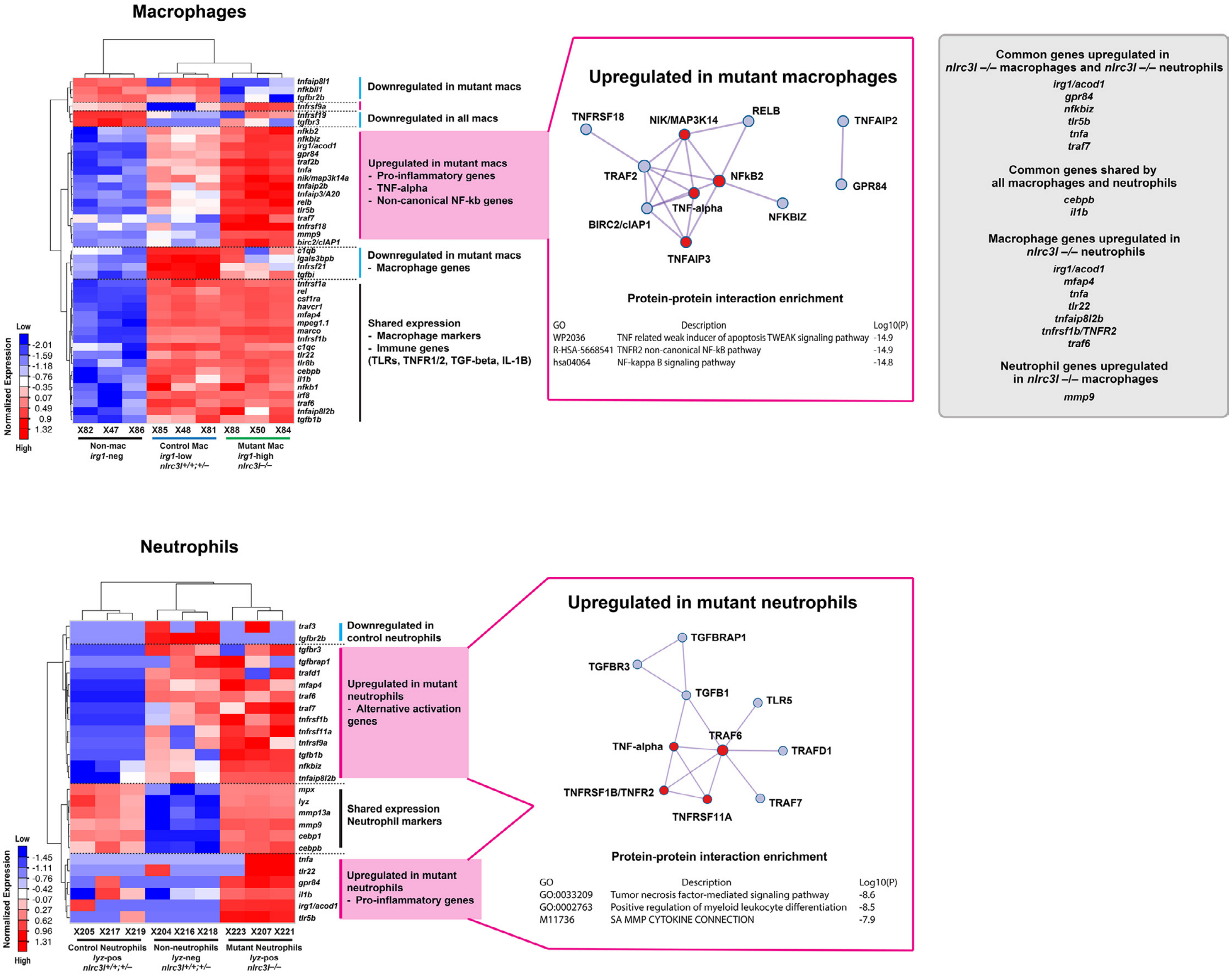

Supplementary Figure 5. Cell-specific transcriptome reveals significant upregulation of non-canonical NF-kB and TNF-alpha pathways in *nlrc3l* mutant immune cells.

Top, a heatmap generated using NG-CHM Builder[1] illustrates normalized expression of a target list of 55 genes, comparing sorted *nlrc3l* mutant and control macrophages alongside non-macrophage cells sorted as *irg1*-negative. Unsupervised hierarchical clustering identified shared gene expressions in control and mutant macrophages as well as significantly upregulated and downregulated genes in *nlrc3l* mutant macrophages compared to controls. Both transcriptome and protein network analyses indicate pronounced elevation of genes in the non-canonical NF-

KB pathway (e.g., *nfkb2*, *traf2b*, *nik/map3k14a*, *relb*) and TNF-alpha pathway (e.g., *tnfa*, *tnfrsf1b*, *tnfrsf11a*, *traf6*) in the mutant macrophages. **Bottom**, using the same analytical approach and target gene list, comparison between mutant and control neutrophils (*lyz*-positive sorted cells) alongside non-neutrophil cells sorted as *lyz*-negative revealed significant upregulation of the TNF-alpha pathway (e.g., *tnfa*, *tnfrsf1b*, *tnfrsf11a*, *traf6*), along with expression of genes typically restricted to macrophages (e.g., *irg1*, *mfap4*, *tnfa*, *tlr22*). Each sample group included three biological replicates. Normalized expression was defined as the z-score of log2-transformed DESeq2 gene counts. Metascape[2] was used for protein-protein interaction enrichment analysis, which uses databases such as STRING, BioGrid, OmniPath, and InWeb_IM. The resulting networks highlight proteins with physical interactions among members of the identified pathways.

Supplementary Figure 6

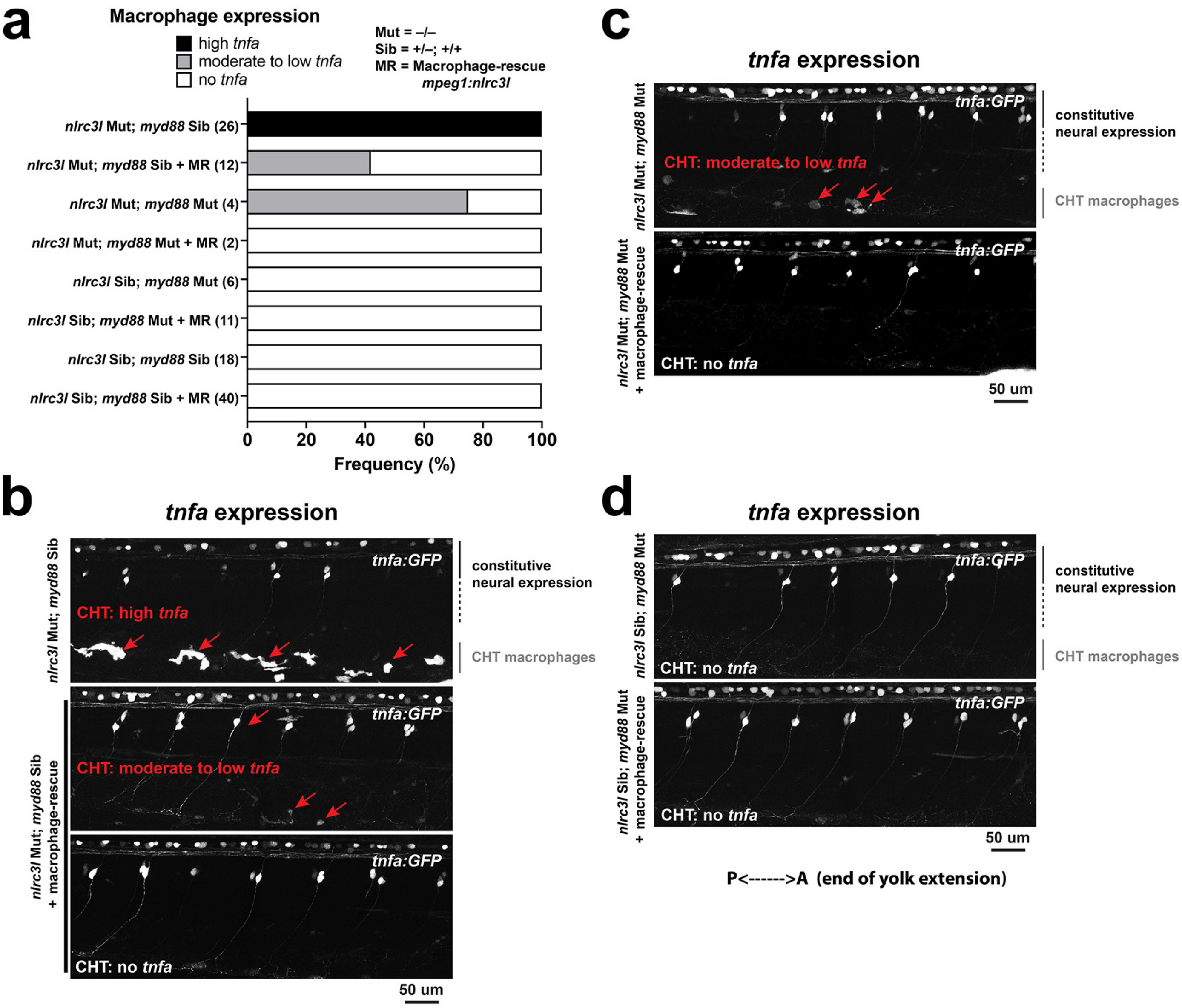

Supplementary Figure 6. Inappropriate *tnfa* induction in *nlrc3l* mutant macrophages is mitigated by *myd88* gene deletion or macrophage rescue, with complete and consistent rescue across animals appearing to require both.

a Percentage of individuals by genotype with high, moderate-to-low, or undetectable *tnfa* expression in macrophages. Numbers in parentheses indicate the sample size (*n*) of individuals analyzed. The animals were derived from incrosses of *nlrc3l; myd88* double heterozygous parents, generating single and double mutants (Mut) alongside their control siblings (Sib). **b-d** Representative images of the *tnfa:GFP* reporter expression analysis for **b** single *nlrc3l* mutants

with or without the macrophage-rescue construct, **c** double *nlrc3l; myd88* mutants with or without the macrophage-rescue (MR) construct, and **d** single *myd88* mutants with or without MR. All images were captured at 3 dpf, with orientation from posterior (left) to anterior (right), starting after the end of yolk extension. All animals were derived from a double *nlrc3l; myd88* heterozygous incross. CHT, caudal hematopoietic tissue.

Supplementary Figure 7

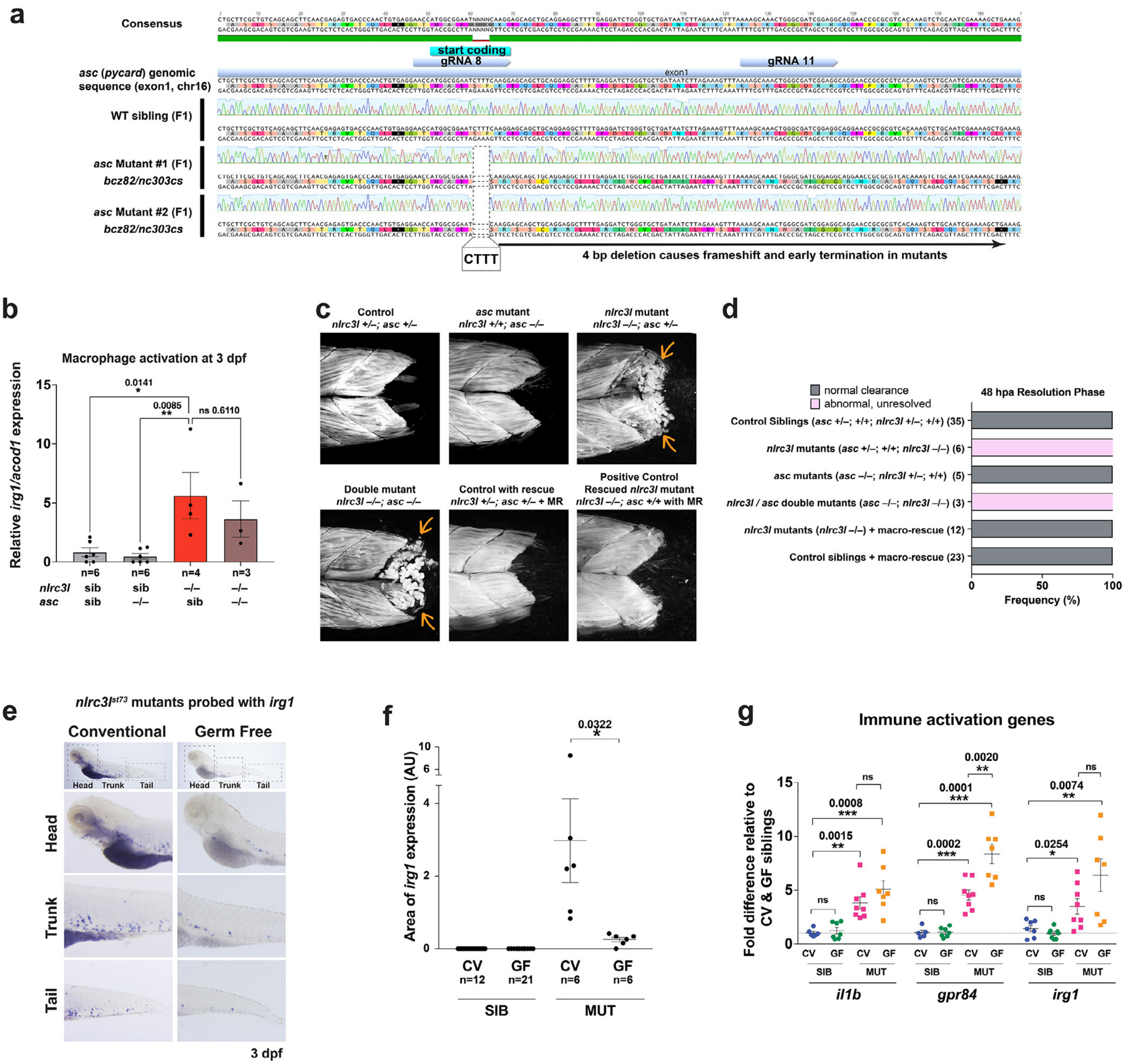

Supplementary Figure 7. While the inappropriate macrophage activation in *nlrc3l* mutants appears to be *asc*-independent, it is partially driven by the commensal microbial environment.

a Generation of a stable frameshift mutation *bcz82* (also known as *nc303cs*) in zebrafish *asc (pycard)* using CRISPR/Cas9 and gRNAs 8 and 11 as shown. Sanger sequencing data shows the mutation causes a four basepair deletion leading to a frameshift and early termination at

residue 4 in the beginning of the coding sequence. Sequencing from two homozygous mutants and one sibling control are shown. This *asc* mutant line was used for the experiments shown in **b-d** and Figure 3 to test possible genetic interaction with *nlrc3l*. **b** Quantification of endogenous *irg1* (also known as *acod1*) transcript levels by qPCR demonstrates aberrantly elevated *irg1* in baseline *nlrc3l* mutants at 3 dpf, as well as in *nlrc3l/asc* double mutants, indicating no evidence of a genetic interaction between *nlrc3l* and *asc*. **c** Functional assessment at 48 hours post-amputation (hpa) shows comparable persistence of unresolved muscle tissue (orange arrows) in *nlrc3l* single and *nlrc3l;asc* double mutants, whereas controls exhibit effective clearance of damaged muscle, further supporting an *asc*-independent role for *nlrc3l.* Macrophage-specific rescue of *nlrc3l* expression, which restores normal repair, was included as a positive control. Images show trunk skeletal muscle by phalloidin staining. MR, macro-rescue achieved by *mpeg1:nlrc3l* transgenic expression. **d** Quantification of phenotypic outcomes across genotypes is presented in the accompanying bar chart. **e** Whole mount RNA in situ hybridization using an anti-sense *irg1* probe shows germ-free *nlrc3l* mutants appear to have less *irg1* expression throughout the body (head, trunk, tail). **f** Quantification of the *irg1* RNA in situ data shows a significant rescue of the detected areas of the *irg1* signal. *n* represents number of embryos analyzed. **g** qPCR analysis of immune activation genes at 3 dpf comparing conventionally raised (CV) and germ-free (GF) animals across genotypes. Overall, the *nlrc3l* mutants had higher expression of immune activation genes regardless of how they were raised compared with control siblings, suggesting that removing microbes did not eliminate the inflammatory phenotype. GF *nlrc3l* mutants appear to have higher expression of all genes but only *gpr84* at a statistically significant level. Due to the lack of spatial information by qPCR and possible transcriptional regulatory mechanisms, the observed reduction of *irg1* activation in GF *nlrc3l* mutants by RNA in situ may be missed by qPCR analysis. SIB, *nlrc3l* +/+ and *nlrc3l* –/– embryos; MUT, *nlrc3l* –/– mutants; CV, conventionally raised; GF, germ-free. AU, arbitrary units. Statistical significance was determined using two-tailed student's t-test.

Supplementary Figure 8

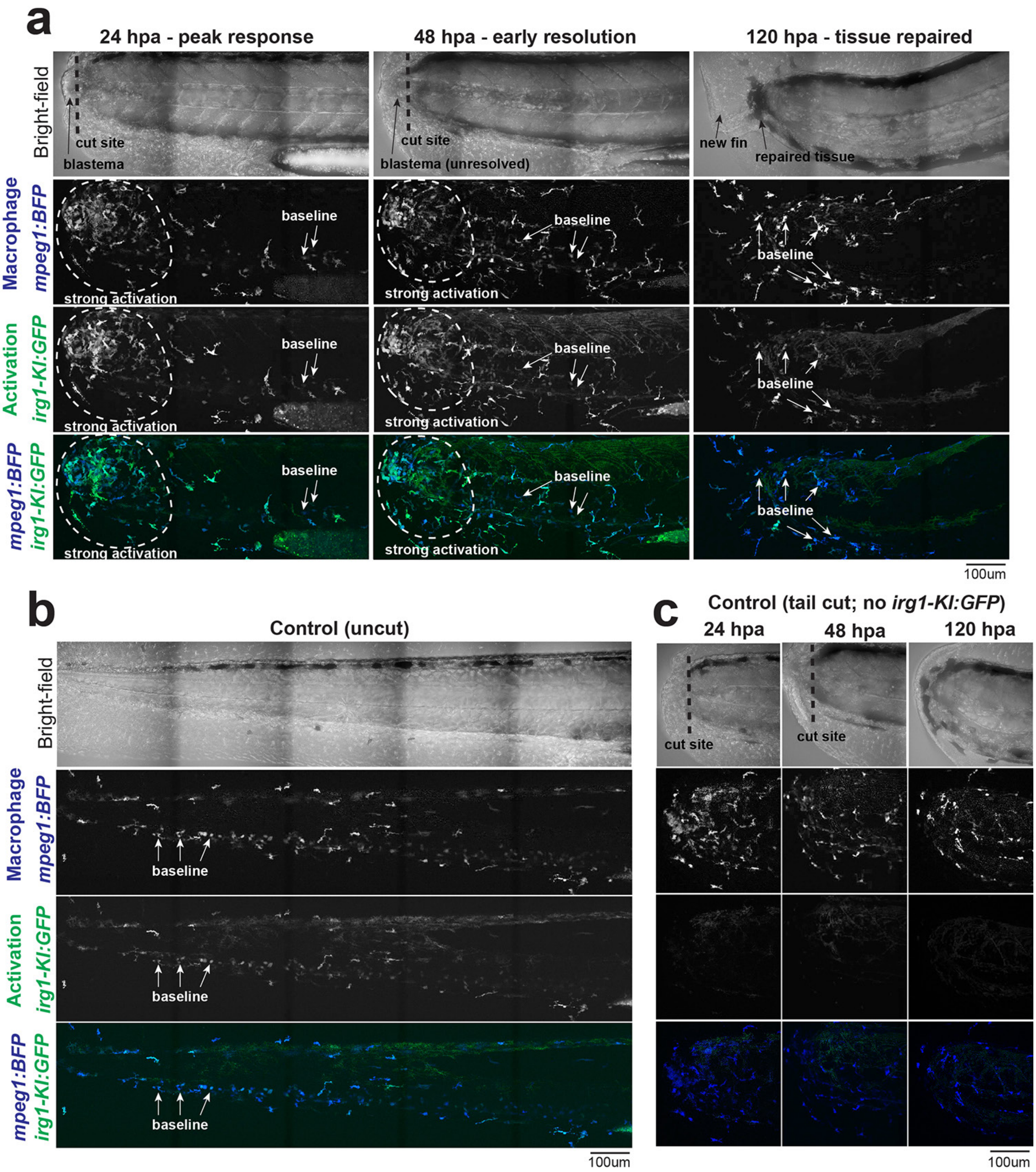

Supplementary Figure 8. Dual labeling of macrophages and their activation state using the *mpeg1* reporter combined with the GFP knock-in allele of *irg1* effectively marks injury-activated macrophages.

a Brightfield images are shown alongside single-channel GFP or BFP and merged channel images, illustrating differences in activation at the cut site versus baseline *irg1* levels in steady state macrophages. The images capture peak response at 24 hpa, early resolution at 48 hpa, and tissue repair by 120 hpa. The cut site is indicated by a dotted line, with the unresolved blastema visible in the brightfield. **b** Uncut control images show no induction of *irg1-KI:GFP* while all macrophages are shown to express *mpeg1* and low *irg1*. **c** As an additional control, tail-truncated embryos expressing only the macrophage reporter show no GFP expression, but exhibit macrophage distribution consistent with an acute injury response.

Supplementary Figure 9

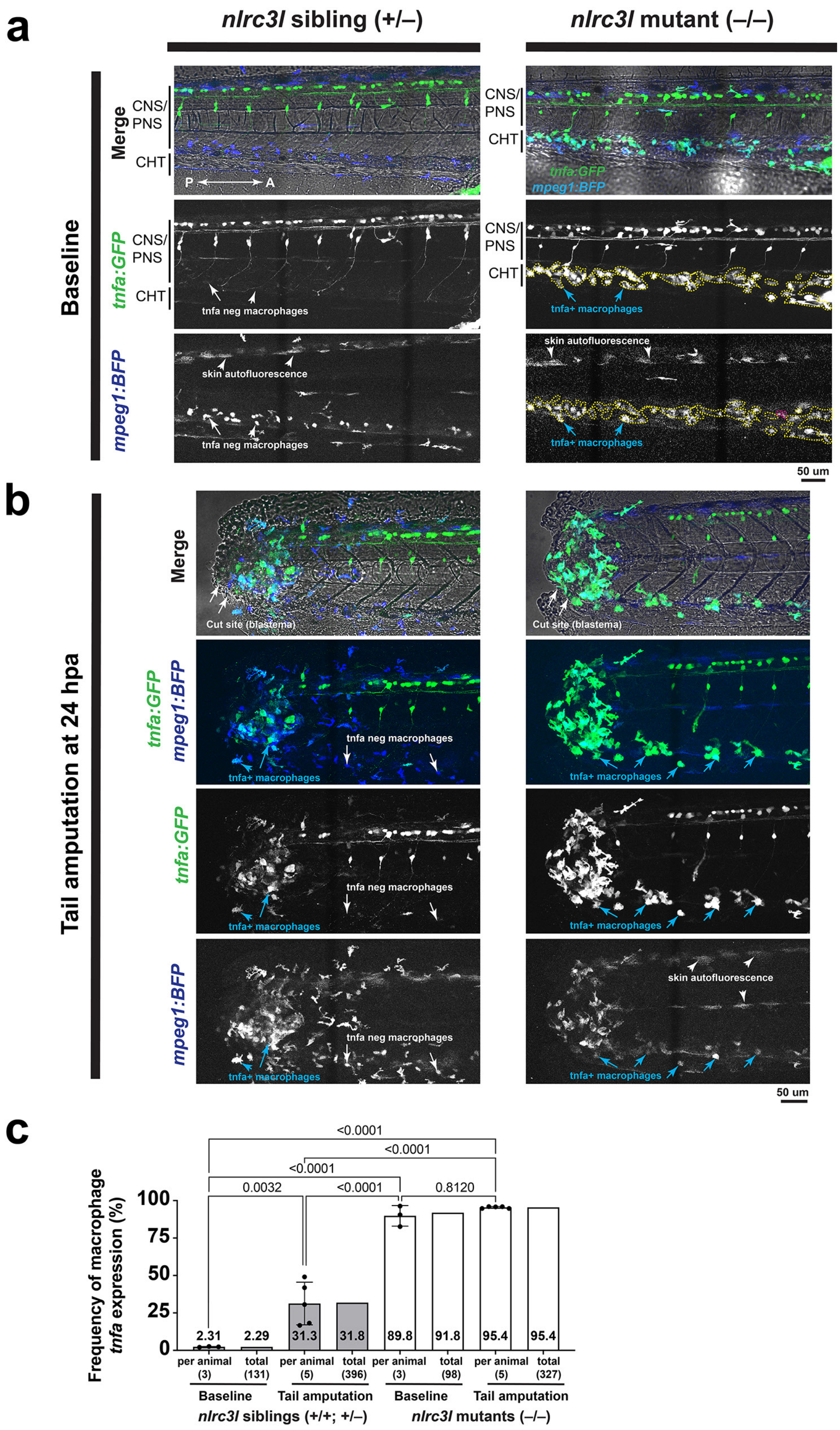

Supplementary Figure 9. Auto-inflammatory *nlrc3l* mutant macrophages aberrantly express *tnfa* at baseline and maintain high *tnfa* expression throughout injury response.

a At baseline, *tnfa* is absent in *nlrc3l* sibling (heterozygous and wild-type) macrophages (white arrows), but is abnormally expressed by nearly all *nlrc3l* mutant macrophages (blue arrows). *mpeg1* marks all macrophages. *tnfa* is constitutively expressed in CNS and PNS neurons, but not normally expressed by macrophages (white arrows) in the CHT (caudal hematopoietic tissue) in the absence of any immune challenge. Skin autofluorescence in the BFP channel is indicated by arrowheads. **b** At 24 hpa, almost all *nlrc3l* mutant macrophages express high *tnfa* levels regardless of location, whereas only a subset of control macrophages post-injury at the cut site highly express *tnfa*. **c** Quantification of macrophage *tnfa* expression at baseline and after tail amputation at 24 hpa. Each genotype and condition category is represented by two plots: one indicated as "per animal" shows data points representing the percentage of *tnfa*+ macrophages from each individual embryo, and the other indicated as "total" depicts the percentage of *tnfa*+ from the total number of macrophages analyzed across all embryos. Statistical significance was determined by one-way ANOVA followed by multiple pairwise comparisons. The number in parentheses represents the sample size (*n*) of independent animals or cells, and the number in each bar graph shows the percentage of animals or cells expressing *tnfa*. Plot shows mean values +/- SD.

Supplementary Figure 10

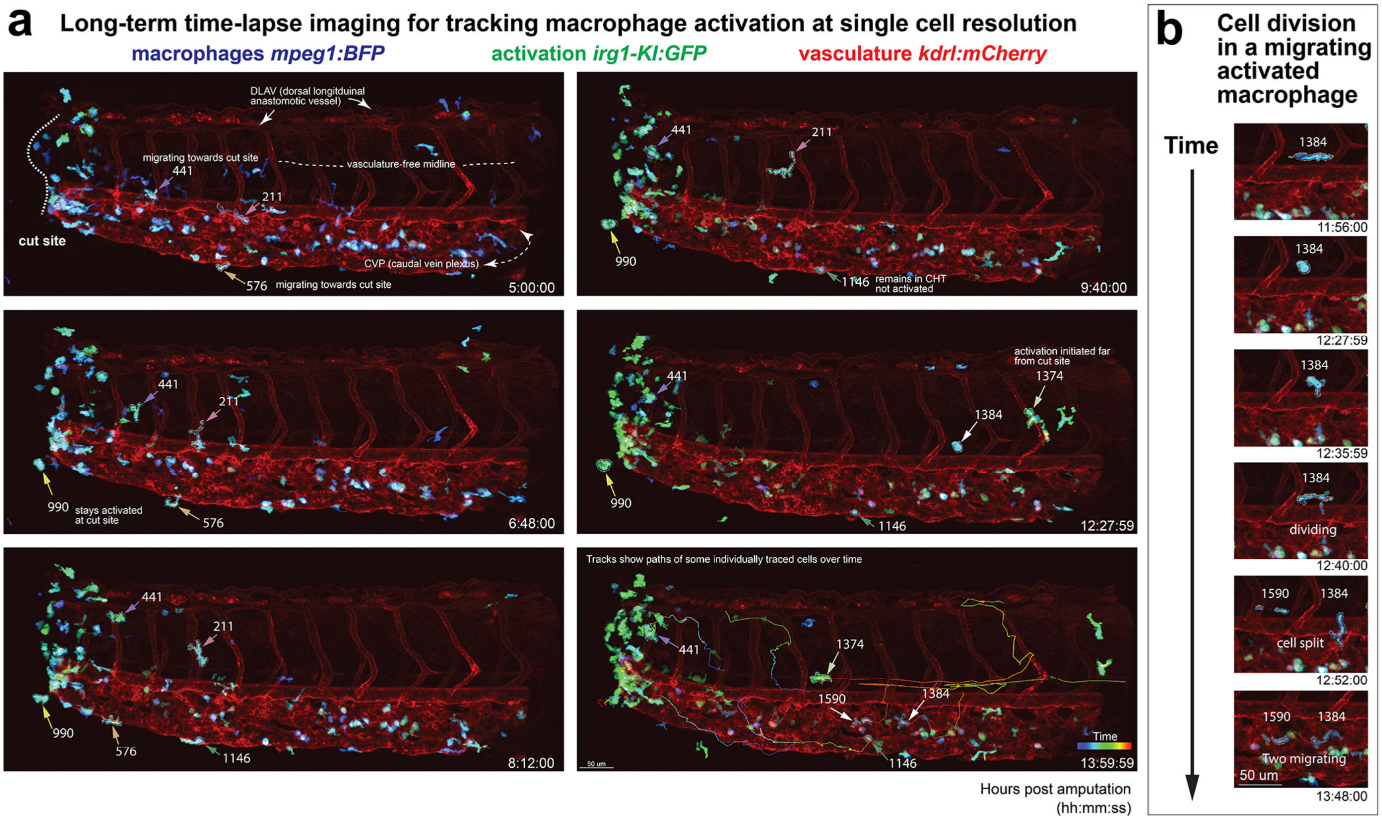

Supplementary Figure 10. Continuous long-term time-lapse imaging of macrophage response during acute injury response provides spatiotemporal dynamics of macrophage activation at a single-cell resolution.

a Visualization of macrophage cellular responses starting at 5 hpa in double transgenic zebrafish with the combined macrophage (*mpeg1*+) and vasculature (*kdrl*+) reporters in the GFP knock-in allele of *irg1*. The spatiotemporal dynamic changes in *irg1* expression and macrophage cellular behavior relative to their position from the cut site can easily be seen. Over time, there is an increase in *irg1* expression, indicated by a shift from blue to brighter green cells, though some cells (e.g., 1146) maintain low baseline *irg1* expression. Many macrophages migrate toward the cut site, noticeably outside the vasculature, with some exhibiting high *irg1* expression prior to reaching the cut site (e.g., 1374). Few cells are individually traced and annotated with numbers and colored arrows; the tracks are shown in the final panel. Time-lapse imaging was performed using a 40x objective as a multi-tile z-stack every 4 minutes for over 10 continuous hours. **b** Migrating macrophages toward the cut site can also be observed to undergo cell division. This

process involves significant morphological changes, with cells transitioning from a stellate shape to a rounded form during mitosis and returning to a migratory stellate cell appearance after division. Scale bars represent 50 μm.

Supplementary Figure 11

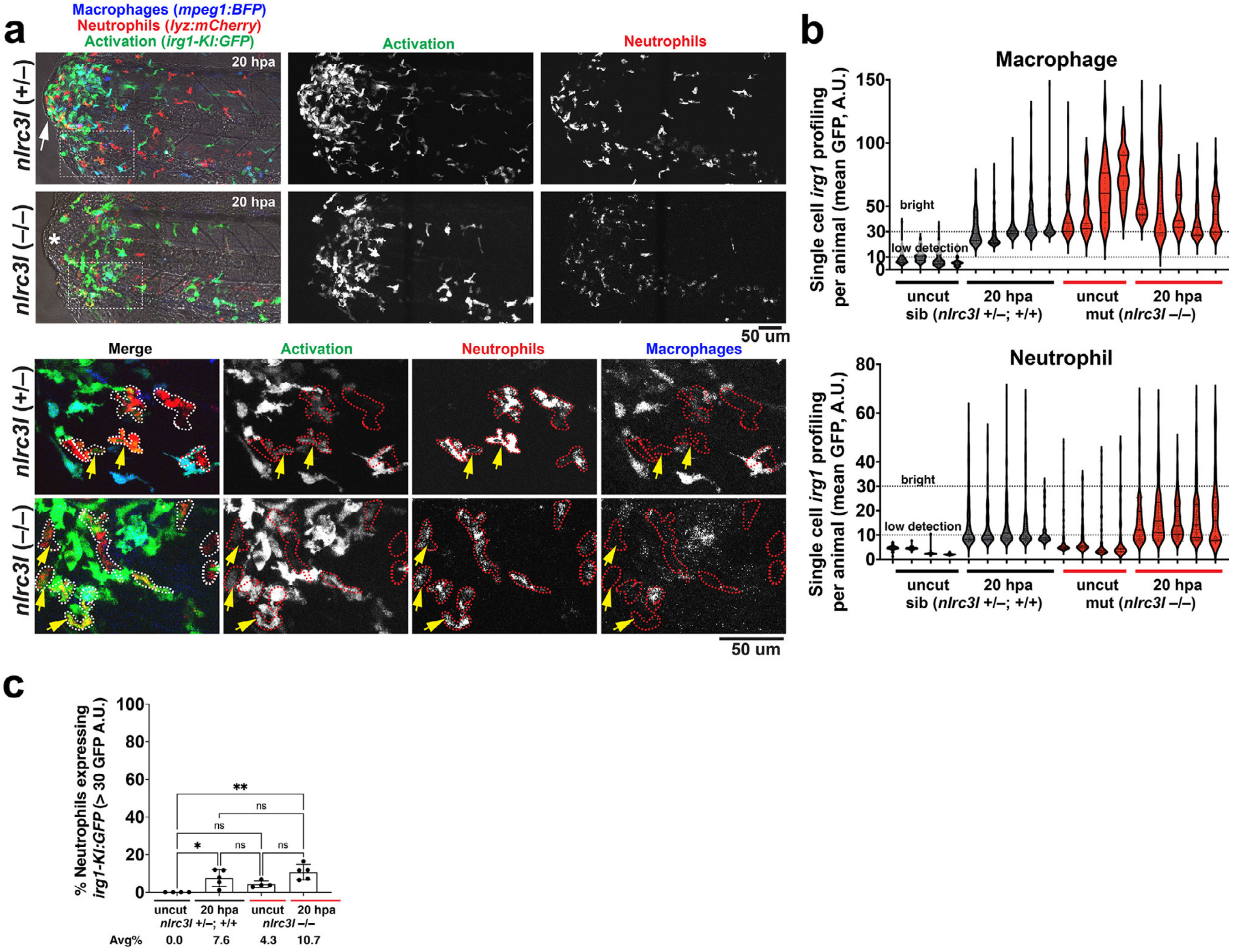

Supplementary Figure 11. ***nlrc3l*** **mutants have unusual profiles of** ***irg1*** **expression in macrophages and neutrophils, correlating with impaired injury response.**

a In vivo confocal imaging of acute injury at 20 hpa in double transgenic zebrafish with reporters for macrophages (*mpeg1*+) and neutrophils (*lyz+)*, and the GFP knock-in allele of *irg1* shows a notable absence of immune cell cluster at the most distal part of the cut site (asterisk) in mutants, compared to typical clustering observed in controls (arrow). Neutrophils are found to intermix with macrophages at their observed locations. Bottom, zoomed in region as indicated in the dotted line box in top panels showing neutrophils expressing *irg1* as outlined by dotted lines (yellow arrows). *n*= 4 animals per genotype condition. **b** Violin plots display quantifications of single-cell *irg1* levels based on GFP knock-in expression, with data for macrophages (top) and neutrophils

(bottom). Each violin plot represents data from an individual animal. These plots, using the same imaging dataset as Figure 3d for quantification, provide a detailed view of the range of *cvfg f* expression within individuals, including the median, quartiles, minimum and maximum values. The dotted line indicates the GFP fluorescence detection threshold: above 10 is low detection and above 30 is visibly bright GFP. Overall, neutrophil *irg1* expression is significantly weaker and confined to a subset of neutrophils compared with macrophages in *nlrc3l* mutants. **c** Bar chart showing the percentage of neutrophils that would be visibly clear under a confocal microscope (>30 GFP arbitrary units, A.U.). No neutrophils would be labeled at homeostasis in the absence of injury in normal embryos. After injury, a small proportion at 7.6% of neutrophils were positive for reporter expression. Mutant neutrophils can express the reporter at baseline or after injury, but rare (<11 %), and their GFP intensity is significantly lower and discernible from that of macrophages.

Supplementary Figure 12

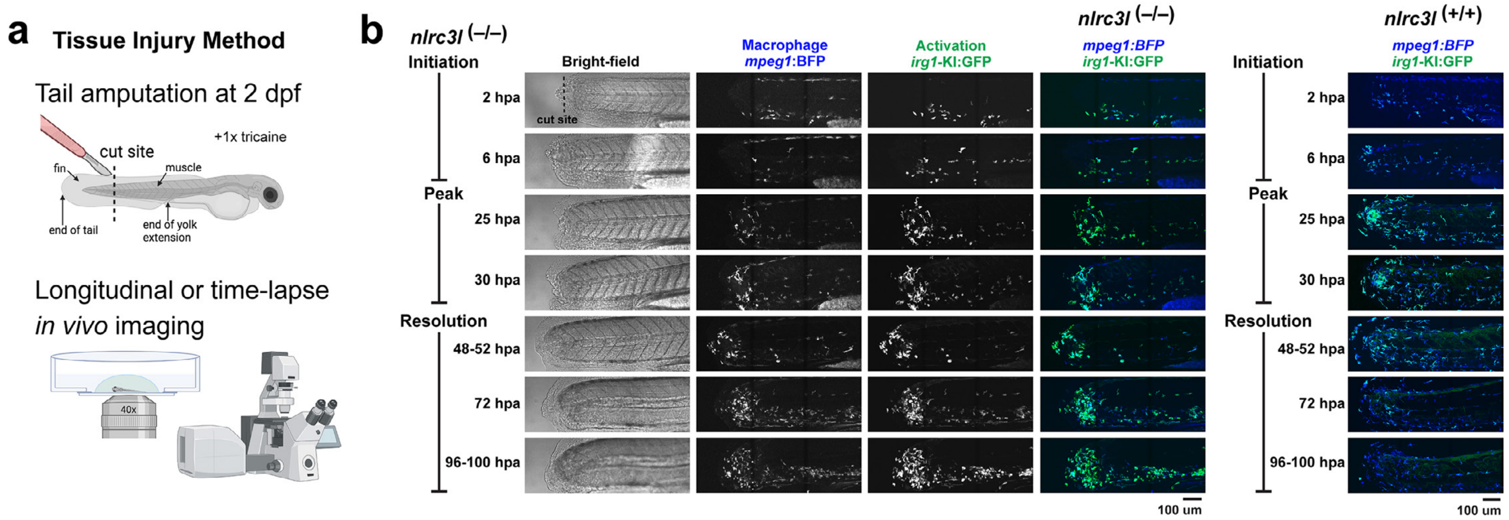

Supplementary Figure 12. Longitudinal analysis reveals altered macrophage distribution and chronically elevated *irg1* expression across all macrophages in *nlrc3l* mutants throughout the acute injury response.

a Schematic of the acute injury model, as described in Figure 4a. Created in BioRender. Shiau, C. (2026) https://BioRender.com/rxau1o9. **b** Time-course images of a representative individual *nlrc3l* mutant, showing real time tracking of the injury response from 2 hpa to 100 hpa. Single-channel images for bright-field, BFP, and GFP are presented alongside the merged channel images for *mpeg1* reporter and GFP knock-in of *irg1*. For comparison, the time series from a wild-type sibling, as shown in Figure 4b, is displayed side-by-side with the *nlrc3l* mutant data.

Supplementary Figure 13

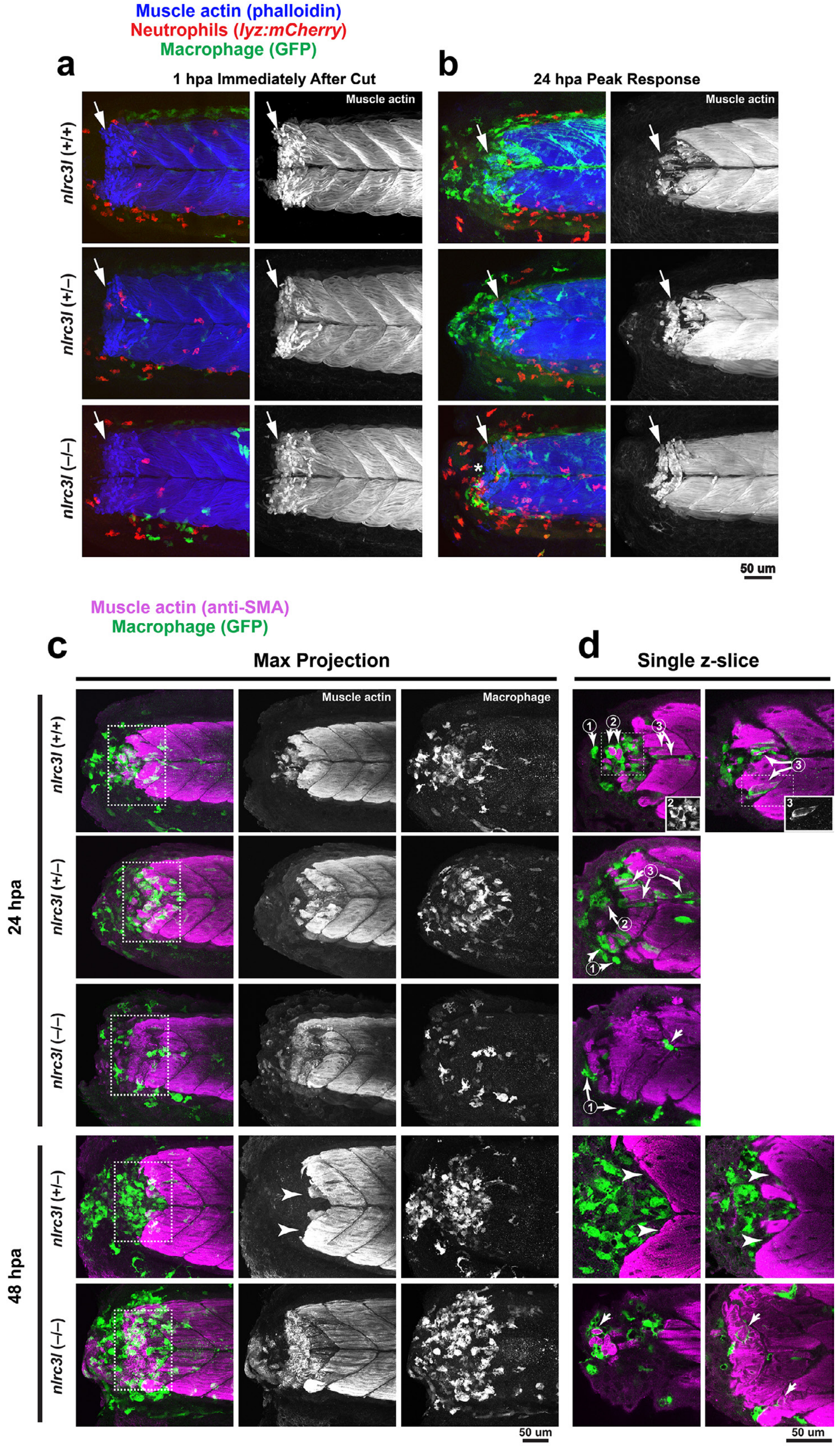

Supplementary Figure 13. Immediate muscle injury was comparable to controls, but injured cells and cellular debris persisted, concurrent with the loss of two functional subtypes in *nlrc3l* mutants.

a-b Immune response to muscle injury was visualized by whole mount IHC soon after operation within 1 hpa (**a**) and later at peak inflammation at 24 hpa (**b**) using phalloidin to stain for muscle actin, GFP antibody detection of *irg1-KI:GFP* for macrophages, and *lyz:mCherry* for neutrophils. The pattern of the immune cells and injured muscle was similar across all genotypes at 1 hpa. Although neutrophil recruitment was comparable between *nlrc3l* mutants and siblings, the mutants had significantly fewer macrophages at the injury site (asterisk) by 24 hpa. This coincided with a more densely packed injured muscle tissue at the damage site (arrow), in contrast to siblings, where macrophages were actively clearing damaged myocytes and leaving gaps behind (arrow, also shown in **c-d** as indicated by subtypes 2 and 3). **c-d** Maximum projection of a confocal z-stack (**c**) shows co-staining of injured muscle using anti-SMA (smooth muscle actin) and macrophages expressing *irg1-KI:GFP* using anti-GFP at 24 hpa or 48 hpa, along with one to two single z-slices (**d**) from the dotted box region of the z-stack, providing a more detailed view of cellular interactions. Inset in the single z-slice panel shows the GFP channel alone from the dotted box region. Three functional macrophage subtypes (1, 2, and 3) are labeled. *nlrc3l* mutants had significantly fewer recruited macrophages at the cut site, only of subtype 1 (arrows), with subtypes 2 and 3 absent. By 48 hpa, macrophage engulfment of muscle fibers in *nlrc3l* mutants was rare and morphologically abnormal (arrows), whereas siblings had cleared debris and formed a clean tissue border (arrowheads). The data for 48 hpa has been reproduced from Figure 4 to allow a direct comparison with 24 hpa.

Supplementary Figure 14

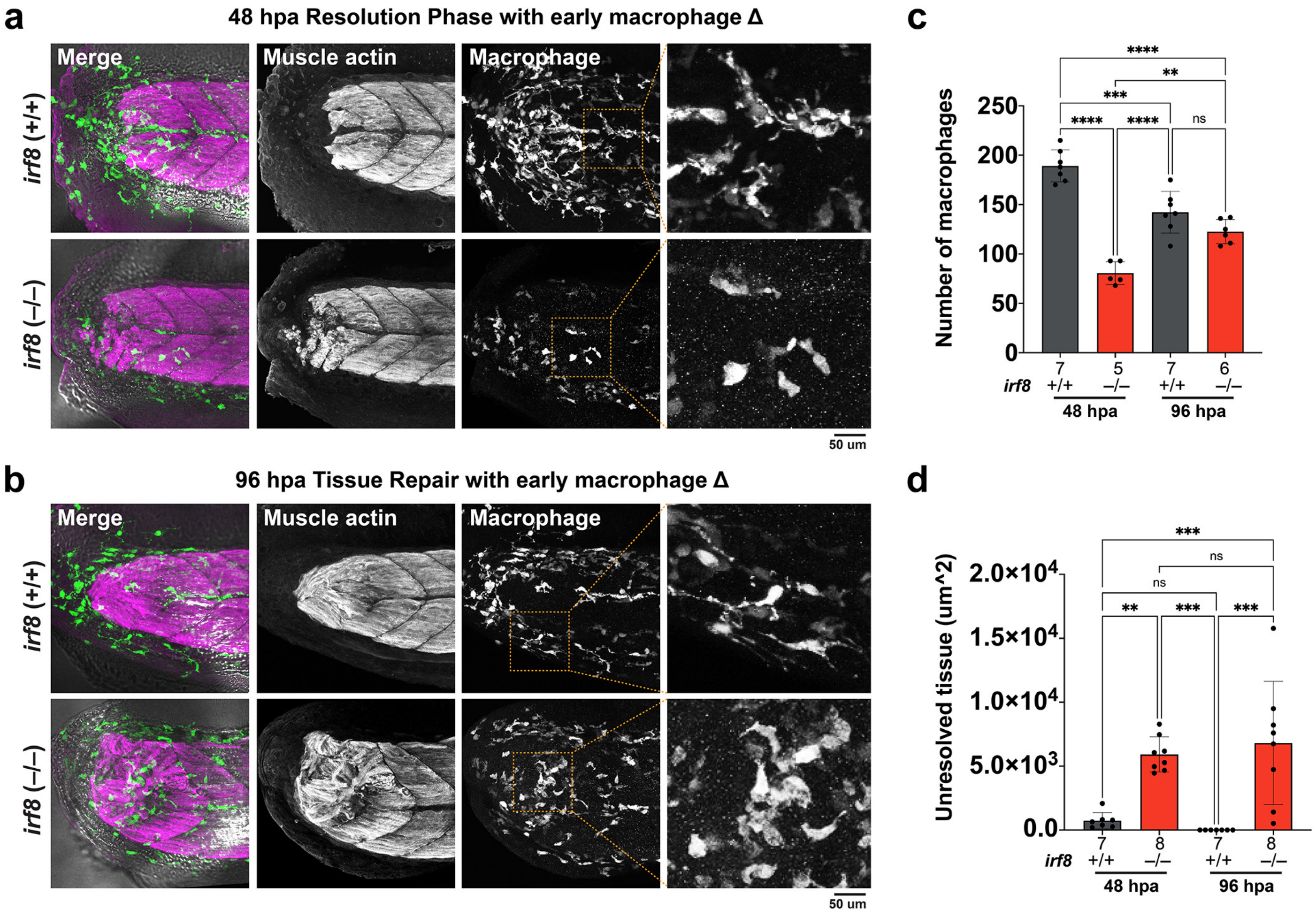

Supplementary Figure 14. Depleting macrophages early in *irf8* mutants leads to impaired acute muscle injury resolution and repair, akin to auto-inflammatory *nlrc3l* mutants.

a-b Whole-mount IHC analysis using anti-SMA to stain muscle actin and anti-GFP to visualize macrophages via *mpeg1:GFP*. The images show inadequate clearance of damaged myocytes at 48 hpa (**a**) and unrepaired muscle tissue at 96 hpa (**b**) in macrophage-depleted *irf8* mutants, despite significant injury-induced macrophage recovery. Merged images are displayed alongside their corresponding single-channel images. A magnified view of the dotted box region shows macrophage morphological differences. **c** Quantification of macrophage numbers in *irf8* mutants compared to control siblings in the region as shown in the images at 48 hpa and 96 hpa. **d** Quantification of unresolved muscle tissue area in *irf8* mutants versus control siblings at 48 hpa and 96 hpa. Each data point represents an individual embryo. Statistical significance was

determined using one-ANOVA followed by multiple comparisons. *n*, number of animals analyzed is shown below each bar. ****, $p<0.0001$; ***, $p<0.001$; ** $p<0.01$; ns, not significant.

Supplementary Figure 15

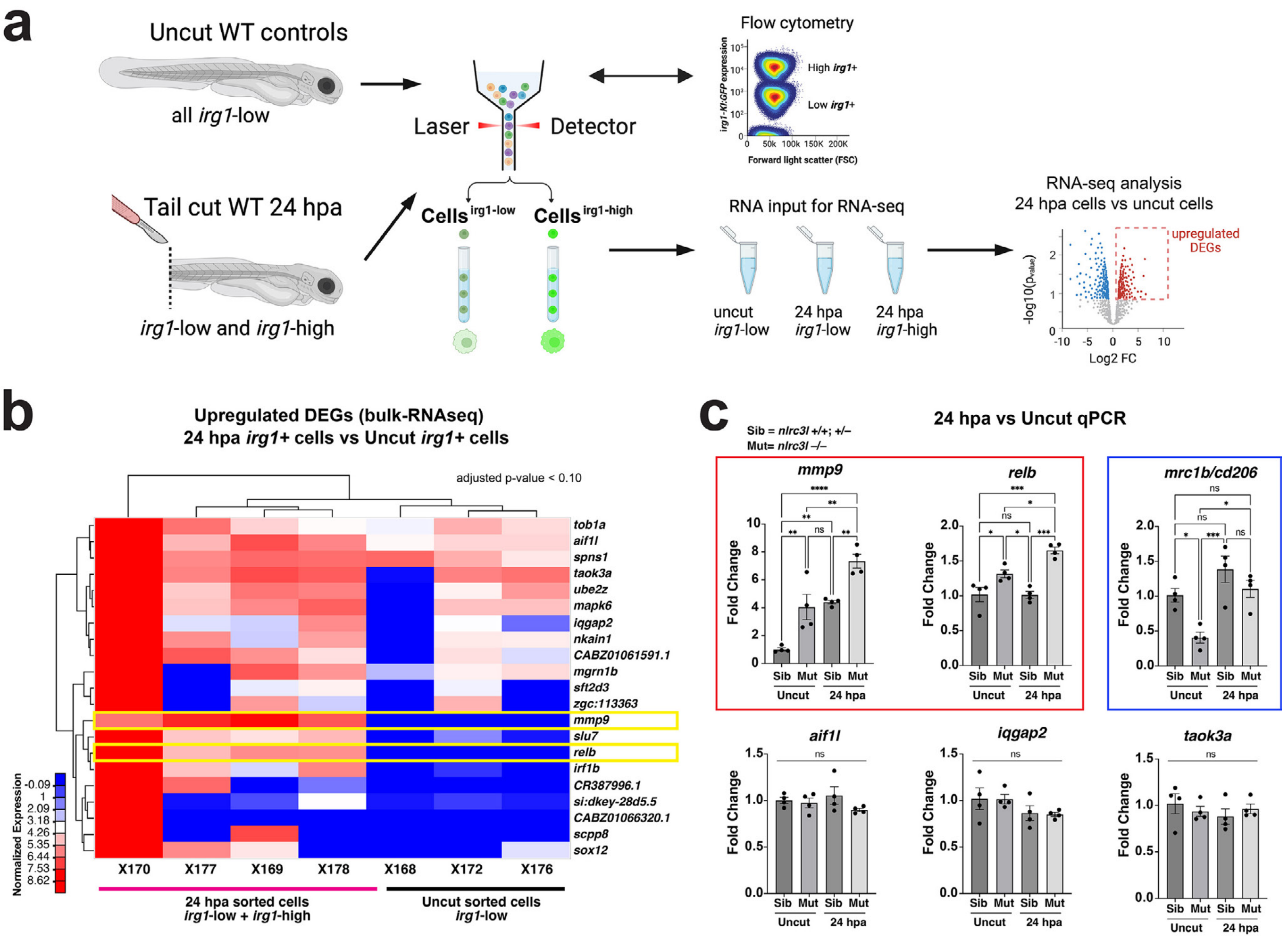

Supplementary Figure 15. Identification of injury-induced macrophage genes by RNA-seq analysis of FACS-sorted macrophages at 24 hpa reveals candidate molecular factors responsible for deficient macrophage functions in *nlrc3l* mutants.

a Diagram of the experimental setup for isolating macrophages by FACS from zebrafish embryos, either uncut controls or 24 hours after tail amputation (performed at 2 dpf). Embryos with the *irg1* GFP knock-in allele were sorted based on low and high GFP levels, followed by total RNA isolation, bulk RNA-seq, and analysis. Each biological replicate was generated by pooling 25-30 embryos for FACS, with three independent biological replicates collected per condition. The RNA-seq read quality was poor for one sample from both the *irg1*-low and *irg1*-high groups, so the remaining samples were re-classified into one larger group named as “24 hpa Macrophages”. Created in BioRender. Shiau, C. (2026) https://BioRender.com/shied5p. **b** Heatmap showing

hierarchical clustering of normalized expression levels of differentially expressed genes (DEGs) in macrophages at 24 hpa (adjusted p-value < 0.10). Yellow boxes highlight two highly significant upregulated genes in 24 hpa macrophages, which were re-assessed by qPCR in 24 hpa control embryos. **c** qPCR analysis of *nlrc3l* mutants (Mut) and their heterozygous and wild-type siblings (Sib) comparing uncut versus 24 hpa embryos for six selected genes based on their possible function in modulating injury or immune response (five upregulated from RNA-seq in panel **a**, and one downregulated). Genes in the red box showed significantly higher expression in *nlrc3l* mutants, while the blue box highlights a gene previously shown to be downregulated in *nlrc3l* mutants (see Figure 2), but which was upregulated post-injury. Although identified as upregulated DEGs in 24 hpa macrophages, *aif1l*, *iqgap2*, and *taok3a* did not show significant changes in qPCR analysis in either control siblings or *nlrc3l* mutants. Each data point represents an independent biological replicate derived from a pool of three embryos. Four independent biological replicates were conducted for each genotype condition. Statistical significance was determined by one-way ANOVA followed by multiple comparisons. ***, $p<0.001$; ** $p<0.01$; *, $p<0.05$; ns, not significant.

Supplementary Figure 16

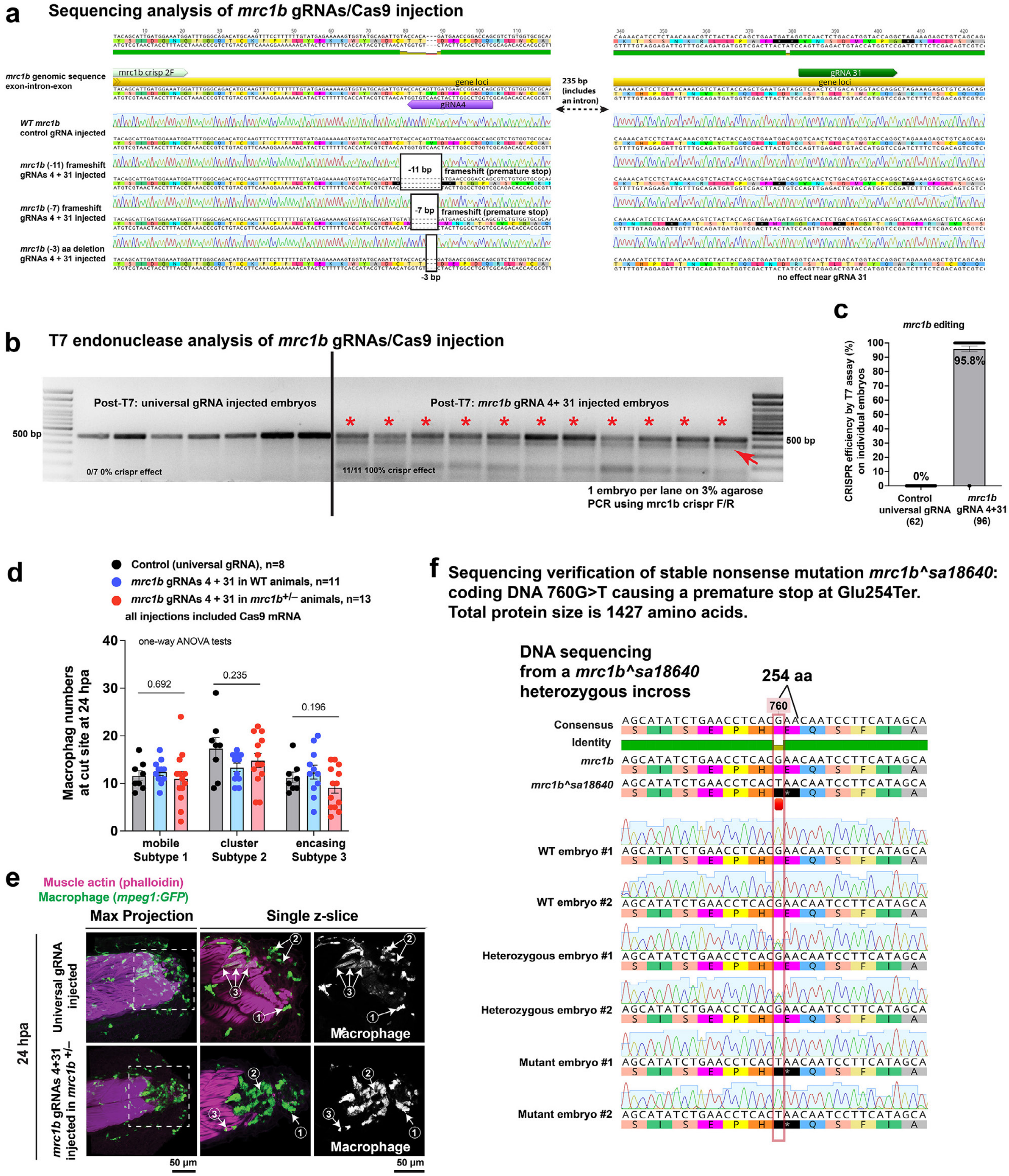

Supplementary Figure 16. Characterization and subtype phenotyping of *mrc1b* editing by CRISPR/Cas9, and verification of *sa18640* mutation in *mrc1b.*

a Sanger DNA sequencing analysis on embryos injected with Cas9 mRNA with *mrc1b*-targeting gRNAs (#4 and #31) compared with universal gRNA/Cas9 injected negative controls showing *mrc1b*-targeting gRNAs induced indels causing frameshifts and premature stops, or amino acid deletion. Sequencing *mrc1b*-targeted embryos suggests CRISPR often mutates only one allele, resulting in heterozygous, non-deleterious outcomes, which is consistent with the partial phenotypes observed. **b** DNA gel electrophoresis shows post-T7 endonuclease assay where only the samples (asterisks) with *mrc1b*-targeting gRNAs resulted in T7 digests as indicated by multiple bands. **c** Quantification of 96 *mrc1b* gRNAs-injected embryos shows 95.8% CRISPR efficiency compared with no editing in universal gRNA/Cas9 injected negative controls (n=62). **d** At 24 hpa, *mrc1b* knockdown zebrafish showed a non-significant but consistent reduction in macrophage subtypes 2 (clustering) and 3 (muscle-encasing), similar to *nlrc3l* mutants with chronic inflammation, supporting *mrc1b* involvement in the mutant phenotype; to improve knockdown efficiency, CRISPR reagents were injected into heterozygous *mrc1b* mutant (*sa18640*) zebrafish, and analysis separating WT siblings revealed a clearer reduction, particularly in muscle-encasing macrophages. **e** Representative maximum projection and a single z-slice from confocal image stacks of a *mrc1b* gRNAs-injected compared with a control injected embryo at 24 hpa showed a reduction in muscle-encasing subtype 3 macrophages. **f** Sanger sequencing confirmed the unverified *sa18640* mutation in *mrc1b*, a G>T substitution at nucleotide 760 causing a premature stop codon at residue 254 of 1427 (red box). The mutant zebrafish line was derived from a large-scale mutagenesis in the UK Sanger Center Zebrafish Project and obtained from ZIRC. The mutation produced Mendelian segregation in the progeny.

Supplementary Figure 17

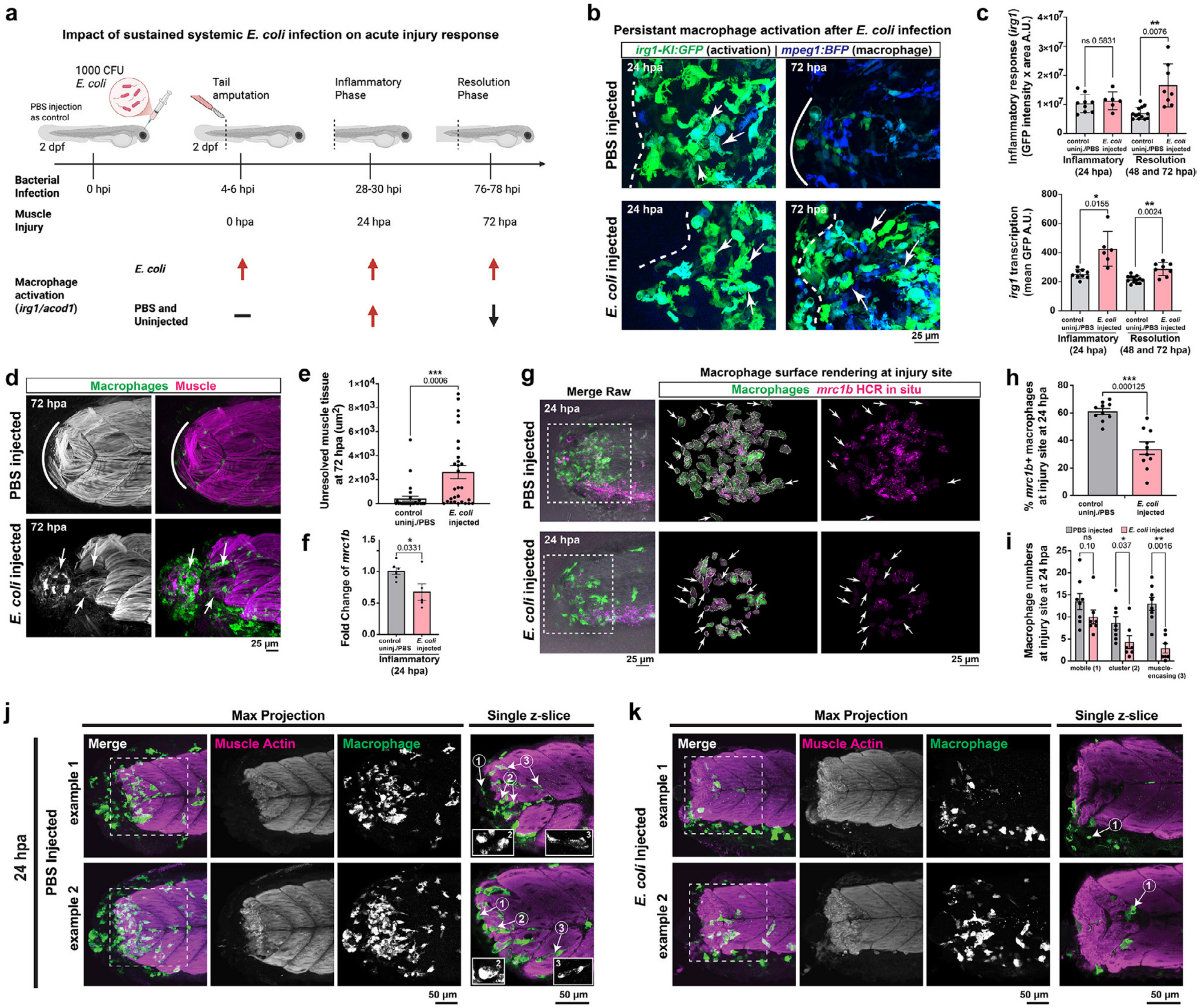

Supplementary Figure 17. Persistent infection induces chronic macrophage activation and impairs tail muscle repair, coinciding with loss of macrophage *mrc1b* and loss of reparative macrophage subtypes 2 and 3 similar to *nlrc3l* mutants.

a Experimental schematic created in BioRender. Shiau, C. (2026) https://BioRender.com/bfa2kgi shows systemic *E. coli* infection induces chronic macrophage activation, marked by sustained *irg1-KI:GFP* expression. Timeline indicates bacterial infection (hours post-infection, hpi) and tail amputation (hours post-amputation, hpa). Infected embryos show prolonged macrophage activation across the entire repair period, in contrast to transient activation during the inflammatory phase (~24 hpa) in controls.

b Confocal images show persistent *irg1-KI:GFP* reporter expression in macrophages at 72 hpa in embryos infected prior to injury, whereas activation resolves in controls (uninfected).

c Quantification of macrophage activation via *irg1-KI:GFP* expression. Top: inflammatory response measured by the number and intensity of *irg1-KI:GFP*$^+$ cells. Both models show similar activation at 24 hpa, but only infection-primed embryos retain high levels at 72 hpa. Bottom: mean GFP intensity in macrophages is higher in infected embryos than controls at all timepoints of analysis, indicating sustained activation. Sample sizes were *n* = 9, 6, 13, and 8 animals per condition, listed in the same order as shown in the plots. Two-tailed Welch's t-test was used to determined statistical significance.

d–e Confocal imaging (**d**) and quantification (**e**) of injury sites at 72 hpa show impaired tail muscle regeneration in infected embryos. Muscle was labeled with phalloidin; macrophages visualized using anti-GFP antibody to label *irg1-KI:GFP*$^+$ cells. Antibody-based GFP detection was used to saturate all GFP$^+$ macrophages, ensuring consistent and accurate cell identification regardless of reporter expression level. Statistical significance was determined using the two-tailed Welch's t-test. *n*= 28 PBS-injected or uninjected (control) and 29 *E. coli* infected animals.

f–g HCR RNA in situ for *mrc1b* transcripts at 24 hpa shows significantly reduced *mrc1b* in infected embryos. *irg1-KI:GFP*$^+$ macrophages were labeled using anti-GFP and 3D-rendered (white surfaces); arrows indicate *mrc1b*-negative macrophages. Statistical significance was determined using the two-tailed student's t-test; n= 6 control PBS-injected and uninjected animals and 5 *E. coli* infected animals.

h Quantification reveals a significant reduction in *mrc1b*$^+$ macrophages at the injury site in infected embryos. *n*= 10 embryos per condition analyzed.

i–k Quantification; *n*= 8 control embryos and 7 *E. coli* infected embryos, (**i**) and representative images (**j–k**) of macrophage subtypes 2 and 3 at 24 hpa show decreased reparative populations in infected embryos. Muscle is shown by phalloidin staining; macrophages are labeled by GFP expression from *irg1-KI:GFP* using anti-GFP staining, and their relative association and location to the muscle cells were used to annotate the subtypes. Each data point represents an individual animal. Statistical significance was determined using a two-tailed Student's t-test: $p<0.05$ (*), $p<0.01$ (**), $p<0.001$ (***); ns, not significant. All error bars show SEM. A.U., arbitrary units.

Supplementary Figure 18

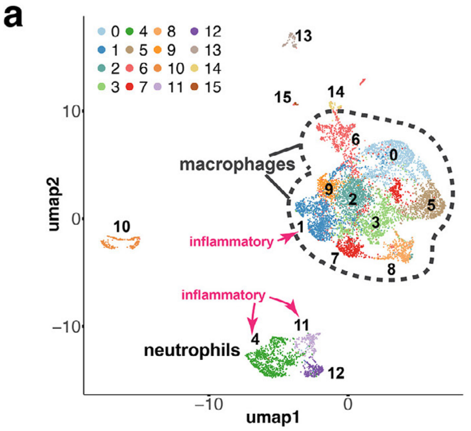

b

Total cell counts per sample after sequencing

Sample	Total Cells
control_uncut_1	662
control_uncut_2	644
control_48hpa_1	907
control_48hpa_2	1059
control_24hpa_1	2032
control_24hpa_2	1701
mut_24hpa_1	1511
mut_24hpa_2	1630

Condition	Total Cells
Control uncut	1306
Control 48hpa	1966
Control 24hpa	3733
Mutant 24hpa	3141

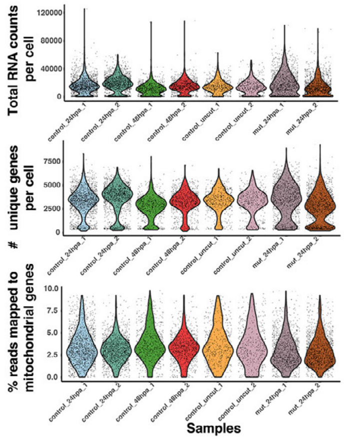

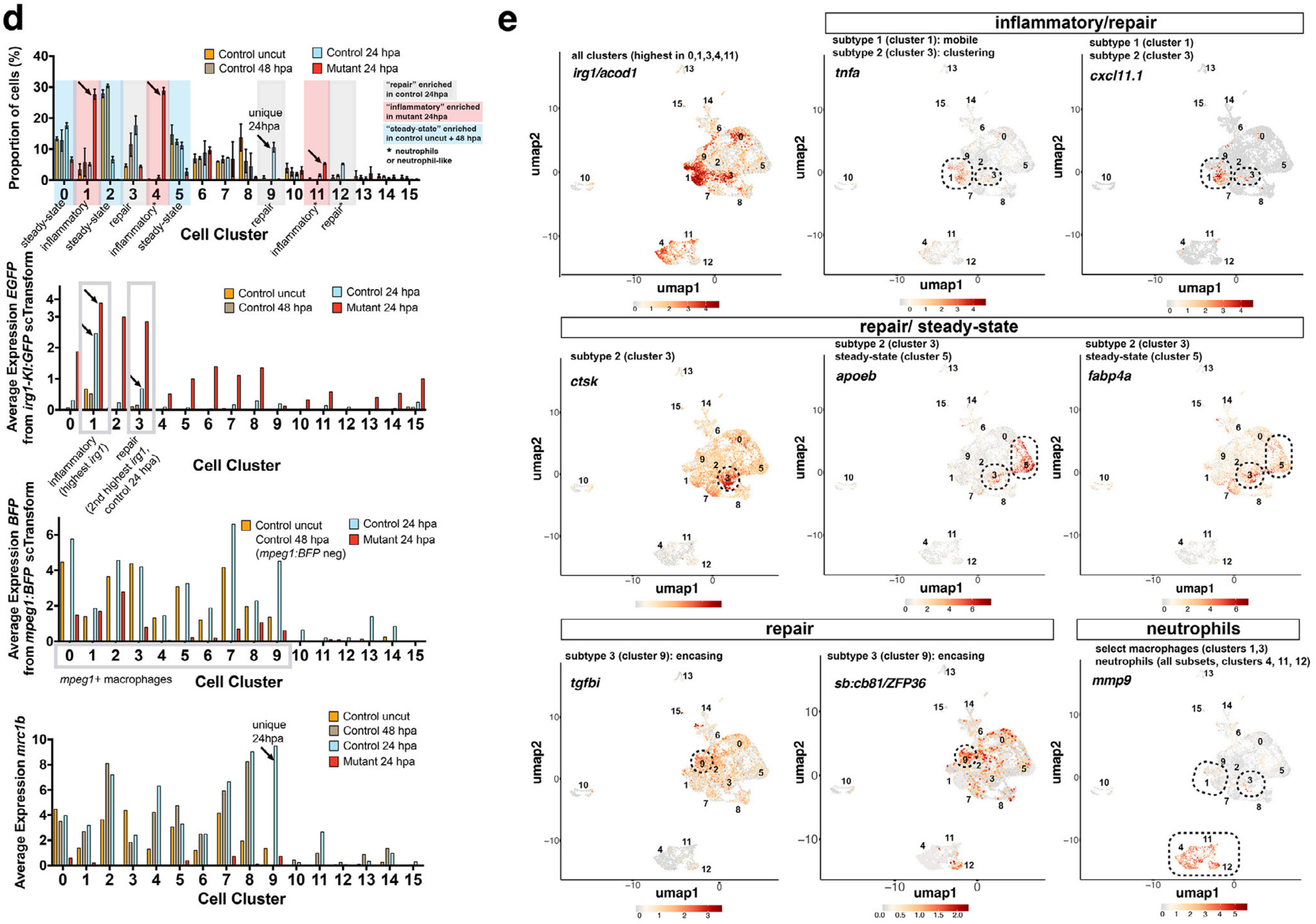

Supplementary Figure 18. Characterization of scRNA-seq data from sorted *irg1*+ cells across conditions define cell clusters and UMAP regions to reveal distinct macrophage and neutrophil subsets.

a UMAP of all sequenced *irg1+* cells across the four experimental conditions, as shown in Fig. 7.
b Table showing total number of cells sequenced per condition.
c Quality control metrics per cell demonstrate consistent sequencing depth and quality across all conditions.
d Bar graphs showing cell proportions and expression levels of EGFP, BFP, and *mrc1b* across all Seurat clusters 0-15. EGFP reflects *irg1-KI:GFP* expression; BFP marks macrophages from *mpeg1:BFP*. These markers help define condition-enriched clusters, e.g., control 24 hpa cells are enriched in cluster 9, while mutant 24 hpa cells are enriched in clusters 1, 4, 11, allowing identification of macrophage subsets based on condition and marker expression.
e UMAPs displaying expression of select genes associated with inflammation, repair, steady-state, and neutrophil identity across all cells. to visualize spatial separation of cells and differential gene expression levels. Gene-enriched regions (excluding *irg1/acod1*) are outlined with dotted borders to highlight the spatial and differential expression patterns.

Supplementary Figure 19

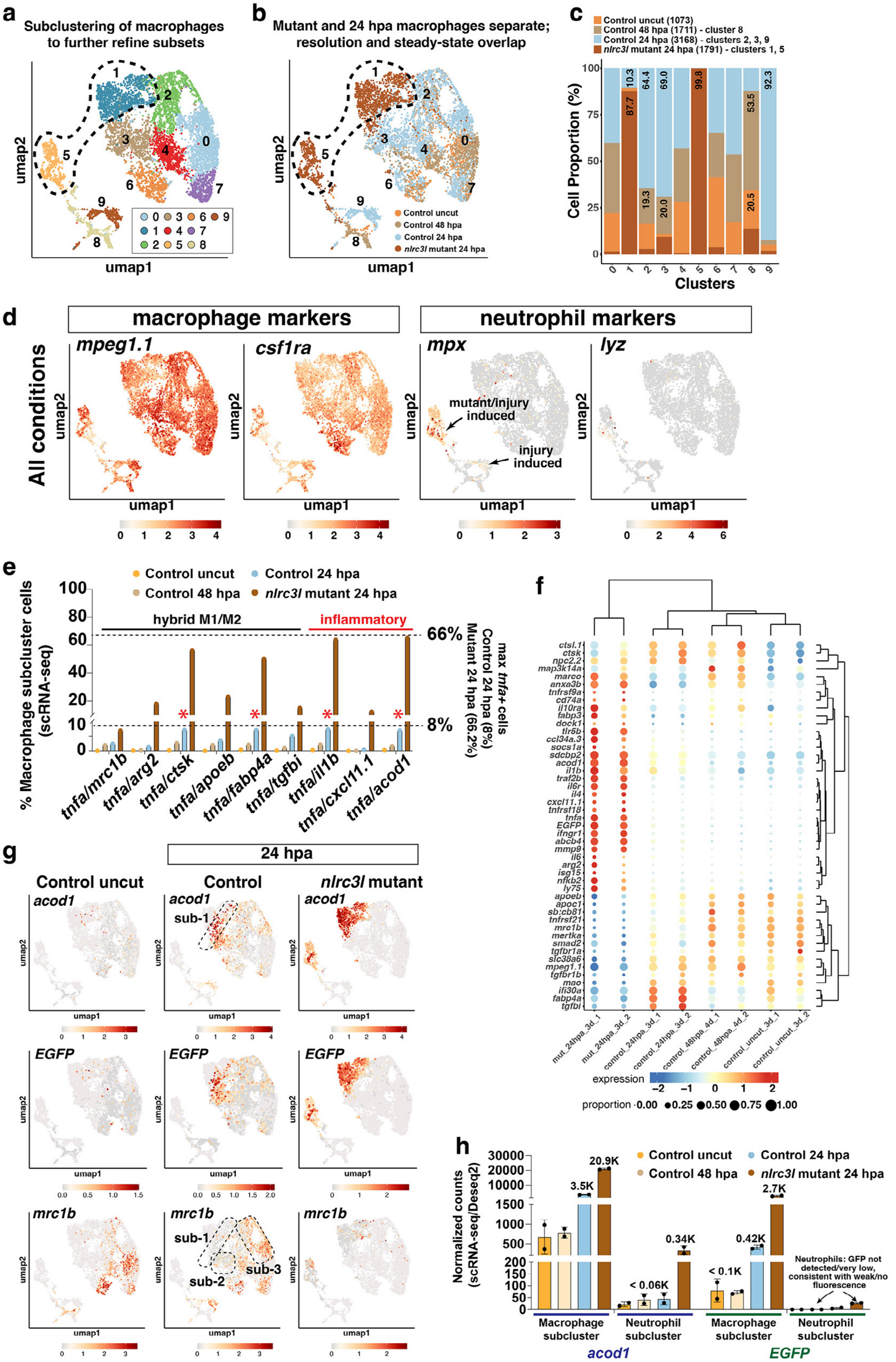

Supplementary Figure 19. Macrophage subclustering reveals compositional and identity changes due to injury response, and further defines the reparative macrophage subsets.

a Subclustering of macrophage-identity cells (from original clusters 0-3 and 5-9, Fig. 7b) yields macrophage-specific UMAPs with subclusters 0-9.
b UMAP colored by experimental condition shows strong overlap between uninjured and control 48 hpa cells, while control 24 hpa and mutant 24 hpa cells separate distinctly.
c Stacked bar plots display subcluster composition per condition, highlighting condition-specific enrichment. The total number of macrophages for each condition is indicated by the numbers in parentheses in the plot legend.
d Marker gene UMAPs confirm macrophage identity across all cells. A small subset of macrophages from mutant or control 24 hpa express low levels of the neutrophil marker mpx, suggesting atypical states yet uncharacterized.
e Percentage of subcluster cells co-expressing *tnfa* and specific markers associated with hybrid M1/M2 or pro-inflammatory states. A larger proportion of mutant macrophages express hybrid M1/M2 markers. Dotted lines indicate the maximum level *tnfa*+ macrophage percentages for visual comparison across 24 hpa conditions.
f Bubble plot with hierarchical clustering shows significantly altered gene expression between control and mutant macrophages at 24 hpa, based on pseudobulk DESeq2 analysis across samples of all conditions. The sample names below the bubble plot denote the genotype and either the injury-response timepoint (24 h or 48 h) or the uncut baseline, with the suffix indicating zebrafish stage (such as 3d for 3 dpf) and biological replicate (such as replicate 1 of 2).
g UMAPs highlight macrophages with the highest *acod1* (also known as *irg1*) and *EGFP* expression across uncut, control 24 hpa, and mutant 24 hpa conditions, defining subset 1 as mutant-enriched and characterized by pro-inflammatory gene expression. Differential *mrc1b* expression across UMAPs further distinguishes the three subsets, with subset 1 (sub-1) showing the lowest *mrc1b* levels, subset 2 (sub-2) displaying intermediate levels, and subset 3 (sub-3) exhibiting higher *mrc1b* expression than subsets 1 and 2 but lower than baseline homeostatic macrophages (derived from uncut controls).
h Bar graphs of normalized gene counts for *acod1* and *EGFP* in macrophage and neutrophil subclusters, highlighting differential expression between these immune cell types. Data relate to Supplementary Fig. 20, which analyzes neutrophil-specific subclusters.

Supplementary Figure 20

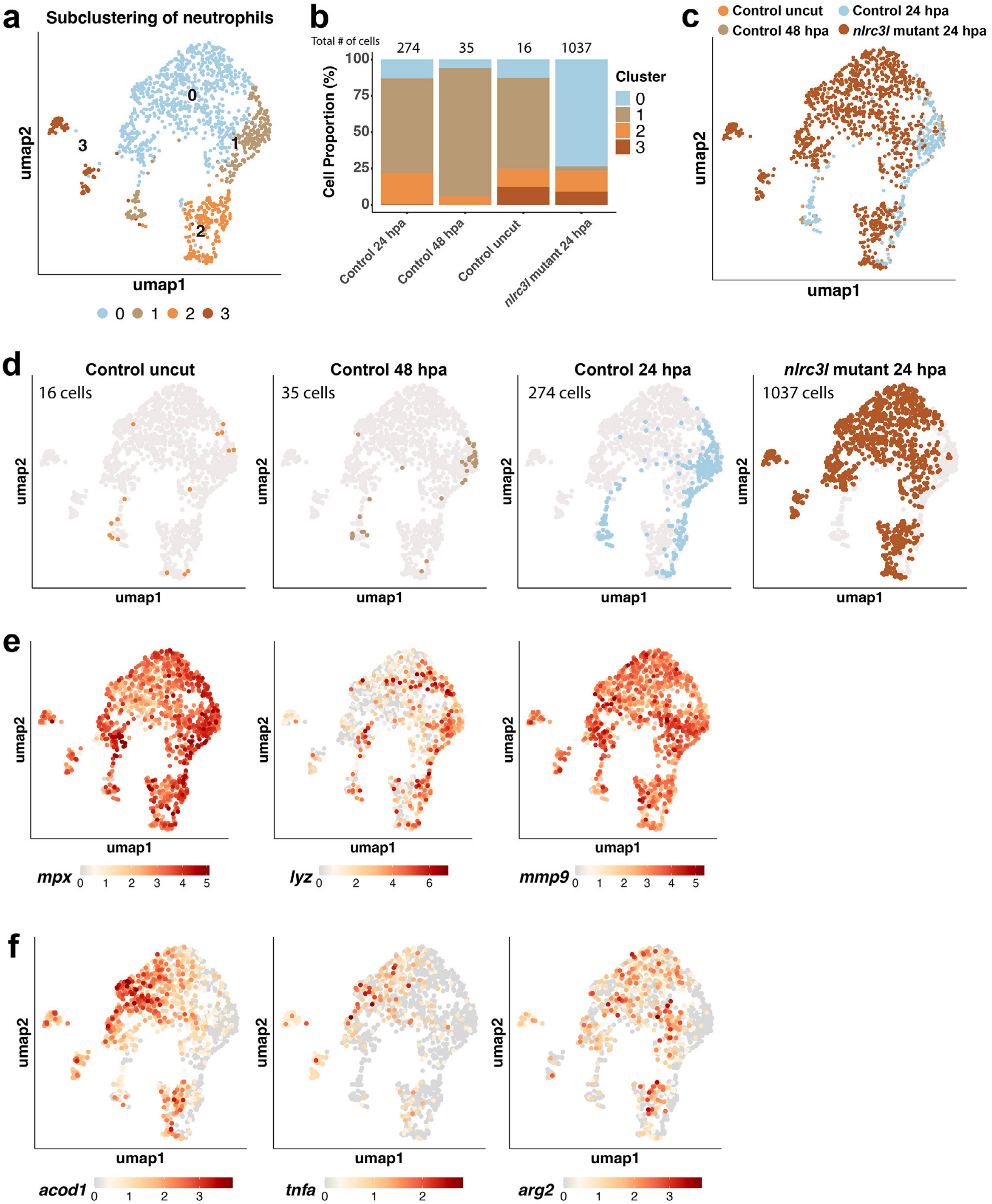

Supplementary Figure 20. Neutrophil subclustering reveals widespread transcriptional activation during the inflammatory phase at 24 hpa, with the majority of cells from *nlrc3l* mutants.

a Subclustering of neutrophil-identity cells (from original clusters 4, 11, and 12, Fig. 7b) yields neutrophil-specific UMAPs with subclusters 0-3.
b Stacked bar plots display subcluster composition per condition, highlighting condition-specific enrichment. The total number of neutrophils for each condition is indicated above each bar graph.
c UMAP colored by experimental condition shows the composition of each condition. The plot clearly shows that the majority of neutrophil-identity cells originate from mutant 24 hpa samples.
d Separate UMAPs by experimental condition are shown to highlight the distinct neutrophils from mutant 24 hpa cells, which is largely separate from the control cells. Control 24 hpa also shows an expansion of neutrophils compared to the baseline (uncut) and resolution phase at 48 hpa.
e-f UMAPs of selected gene markers highlight the distribution of cells expressing neutrophil markers (*mpx, lyz, mmp9*)(**e**), immune activation or pro-inflammatory genes (*acod1, tnfa*) and a typical M2/pro-repair gene (*arg2*)(**f**). All genes are strongly expressed in *nlrc3l* mutant neutrophils at 24 hpa, except for *lyz*, which notably marks primarily control neutrophils.

Supplementary Figure 21

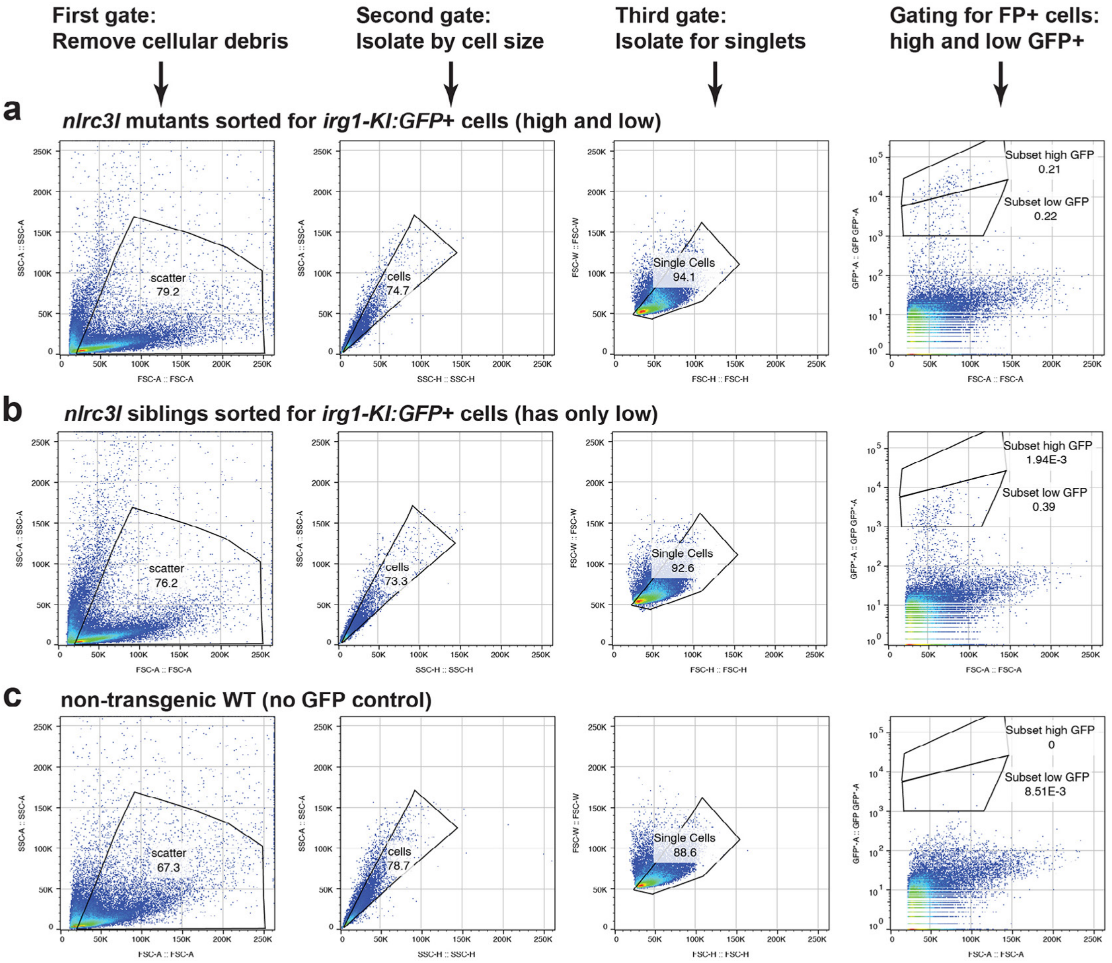

Supplementary Figure 21. Gating strategy for isolating fluorescent protein expressing immune cells from zebrafish embryos using FACS sorting.

The same gating approach was used to isolate macrophages and neutrophils, whereby the first selections are made to eliminate cellular debris and non-singlets followed by a positive selection for fluorescent protein (FP) expression, either for GFP+ cells from *irg1-KI:GFP*+ embryos or mCherry+ cells from *lyz:mCherry*+ embryos, respectively. Diagrams show actual examples of isolating macrophages from **a** *nlrc3l* mutants, **b** control siblings, and **c** non-transgenic wild-type embryos, which served as negative controls. Each sample was a homogenate of 20-50 embryos at 3 dpf. Dissociated *irg1-KI:GFP*+ embryos treated with heat shock to induce cell death showed no GFP+ cells above the gating threshold, while the same homogenate prior to heat shock had abundant GFP+ cells, so all cells/events above the GFP threshold can be considered live cells. Each plot shows a number, which represents the percentage of the total events represented in the depicted gate.

Supplementary References

1 Broom, B. M. *et al.* A Galaxy Implementation of Next-Generation Clustered Heatmaps for Interactive Exploration of Molecular Profiling Data. *Cancer Res* **77**, e23-e26, doi:10.1158/0008-5472.CAN-17-0318 (2017).

2 Zhou, Y. *et al.* Metascape provides a biologist-oriented resource for the analysis of systems-level datasets. *Nat Commun* **10**, 1523, doi:10.1038/s41467-019-09234-6 (2019).
