# Supplementary material for: Chronic macrophage activation derails muscle repair by disrupting mannose-receptor-linked plasticity revealed by endogenous irg1/acod1 tracking
Source: Nat Commun. 2026 Jan 7;17:1466. doi: 10.1038/s41467-025-68204-3 (PMC12886890; doi:10.1038/s41467-025-68204-3)
Supplement: Supplementary file 2 — Description of Additional Supplementary Files [file 41467_2025_68204_MOESM2_ESM.pdf]

Description of Additional Supplementary Files

Title: **Supplementary Data 1**
Description: A list of key resources and reagents used in this study.

Title: **Supplementary Data 2**
Description: Source Data.

Title: **Supplementary Movie 1. Timelapse imaging shows spatiotemporal dynamics of macrophage activation during initial response to acute muscle injury starting from 5 hpa.**
Description: Triple labeled wild-type zebrafish was used to visualize individual macrophage state and behavioral changes during injury response using combined macrophage (*mpeg1:BFP*) and vasculature (*kdrl:mCherry*) reporters in the *irg1-KI:GFP* heterozygous background. Individually tracked cells are labeled by a number and an arrow. Confocal z-stack was captured every 4 minutes with a z-slice depth of 3 um x 38 using a 40x objective. Movie shows imaging data after being processed in Imaris to render macrophages for tracking and remove non-specific skin autofluorescence. Total imaging time shown is 9 hours. Time stamp shows relative time starting from 5 hpa; h:mm:ss. CHT, caudal hemopoietic tissue. See also Supplementary Fig. 10 for additional description.

Title: **Supplementary Movie 2. Timelapse imaging shows immune deactivation and reversal migration during tissue resolution phase.**
Description: Wild-type double transgenic zebrafish was used to track macrophages (*mpeg1:BFP*) and their activation state (*irg1-KI:GFP*) alongside neutrophils (*lyz:mCherry*). Movie shows downregulation of *irg1* in macrophages, most returning to homeostatic levels (appearing mostly BFP+), while they migrate away from the injury site. Confocal z-stack was captured every 4 minutes with a z-slice depth of 3 um x 40 using a 40x objective. Total imaging time was ~ 15 hours. Dotted line marks approximate boundary between muscle and tail fin. Arrows indicate general direction of movement by macrophages. CHT, caudal hematopoietic tissue. Time stamp shows h:mm:ss.

Title: **Supplementary Movie 3. Normal macrophage and neutrophil behavioral response to acute muscle injury was visualized during the peak inflammatory phase at 24 hpa in control *nlrc3l* heterozygote.**

Description: Timelapse imaging of double transgenic zebrafish (*irg1-KI:GFP* for macrophages and *lyz:mCherry* for neutrophils) shows distinct macrophage subtypes (1, 2, 3) as labeled by numbers and arrows based on distinct behaviors: 1: high motility and surveillance (pink arrows), 2: rounded and phagocytic (orange arrows), and 3: stationary and columnar (white arrows and dotted line region) as those expected to encase the injured or repairing myocytes. White line marks end of the tail. Confocal z-stack was captured every 2 minutes with a z-slice depth of 3 um for a total of 120 um using a 40x objective. Total imaging time was 1 hour. Time stamp shows h:mm:ss.

Title: **Supplementary Movie 4. Split-screen movie file corresponding to Supplementary Movie 3, showing macrophage rendering and tracking.**

Description: The left window displays macrophages with their cellular surfaces outlined, color-coded by subtype (white for subtype 1, blue for subtype 2, and red for subtype 3). Macrophage cell trajectories are tracked and color-coded based on time using a heat map. The right window shows the rendering and tracking independently.

Title: **Supplementary Movie 5. Severely deficient macrophage and neutrophil behavioral response to acute muscle injury was observed during the peak inflammatory phase at 24 hpa in auto-inflammatory *nlrc3l* mutants.**

Description: Timelapse imaging of double transgenic zebrafish (*irg1-KI:GFP* for macrophages and *lyz:mCherry* for neutrophils) reveals presence of only subtype 1 (highly migratory, pink arrows) loss of macrophage subtype 2 (phagocytic) and subtype 3 (muscle-encasing). Confocal z-stack was captured every 2 minutes with a z-slice depth of 3 um for a total of 108 um using a 40x objective. Total imaging time was 1 hour. Time stamp shows h:mm:ss.

Title: **Supplementary Movie 6. Split-screen movie file corresponding to Supplementary Movie 5, showing macrophage rendering and tracking in *nlrc3l* mutants.**

Description: The left window displays macrophages with their cellular surfaces outlined, color-coded by subtype (white for subtype 1, blue for subtype 2, and red for subtype 3). Only subtype 1 is observed, so cells are marked by white surfaces. Macrophage cell trajectories are tracked and color-coded based on time using a heat map. The right window shows the rendering and tracking independently.
