# Supplementary material for: Chronic macrophage activation derails muscle repair by disrupting mannose-receptor-linked plasticity revealed by endogenous irg1/acod1 tracking
Source: Nat Commun. 2026 Jan 7;17:1466. doi: 10.1038/s41467-025-68204-3 (PMC12886890; doi:10.1038/s41467-025-68204-3)
Supplement: Supplementary file 11 — Reporting Summary [file 41467_2025_68204_MOESM11_ESM.pdf]

nature portfolio

Corresponding author(s): Shiau, Celia

Last updated by author(s): 12/13/2025

Reporting Summary

Nature Portfolio wishes to improve the reproducibility of the work that we publish. This form provides structure for consistency and transparency in reporting. For further information on Nature Portfolio policies, see our Editorial Policies and the Editorial Policy Checklist.

Statistics

For all statistical analyses, confirm that the following items are present in the figure legend, table legend, main text, or Methods section.

n/a	Confirmed	
☐	☒	The exact sample size (*n*) for each experimental group/condition, given as a discrete number and unit of measurement
☐	☒	A statement on whether measurements were taken from distinct samples or whether the same sample was measured repeatedly
☐	☒	The statistical test(s) used AND whether they are one- or two-sided<br>*Only common tests should be described solely by name; describe more complex techniques in the Methods section.*
☐	☒	A description of all covariates tested
☐	☒	A description of any assumptions or corrections, such as tests of normality and adjustment for multiple comparisons
☐	☒	A full description of the statistical parameters including central tendency (e.g. means) or other basic estimates (e.g. regression coefficient) AND variation (e.g. standard deviation) or associated estimates of uncertainty (e.g. confidence intervals)
☐	☒	For null hypothesis testing, the test statistic (e.g. *F*, *t*, *r*) with confidence intervals, effect sizes, degrees of freedom and *P* value noted<br>*Give P values as exact values whenever suitable.*
☒	☐	For Bayesian analysis, information on the choice of priors and Markov chain Monte Carlo settings
☒	☐	For hierarchical and complex designs, identification of the appropriate level for tests and full reporting of outcomes
☒	☐	Estimates of effect sizes (e.g. Cohen's *d*, Pearson's *r*), indicating how they were calculated

Our web collection on statistics for biologists contains articles on many of the points above.

Software and code

Policy information about availability of computer code

Data collection	Microscopy data were collected from the Nikon A1R+ imaging system as described in methods using the Nikon NIS-Elements acquisition software, and from the stereoscope Leica M165 FC using Leica's Las X software. FACS data and sorted cells were collected from BD FACSMelody Cell Sorter using BD FACSChorus Software. qPCR was collected from the QuantStudio 3 Real-Time PCR System (Applied Biosystems) using QuantStudio Real-Time PCR software. RNA high-throughput sequencing data were collected on Illumina NovaSeq6000 S2, NovaSeq6000 SP, or NovaSeq X Plus using their associated software from Illumina.
Data analysis	Microscopy imaging data that were further processed for quantifications were analyzed either using ImageJ Fiji version 2.9.0 or Imaris 10.1.1.software (Bitplane Oxford) as described in the Methods. Prism 10 was used for statistical tests and plotting data. FlowJo 10.8.2 was used to analyze FACS data.

For manuscripts utilizing custom algorithms or software that are central to the research but not yet described in published literature, software must be made available to editors and reviewers. We strongly encourage code deposition in a community repository (e.g. GitHub). See the Nature Portfolio guidelines for submitting code & software for further information.

Data

Policy information about availability of data

All manuscripts must include a data availability statement. This statement should provide the following information, where applicable:

- Accession codes, unique identifiers, or web links for publicly available datasets
- A description of any restrictions on data availability
- For clinical datasets or third party data, please ensure that the statement adheres to our policy

Data generated in this study are provided in the publication, Supplementary Information and Source Data Files. RNA sequencing data is freely available at the GEO repository as GSE283438 (https://www.ncbi.nlm.nih.gov/geo/query/acc.cgi?acc=GSE283438) for bulk RNA-seq, and GSE301079 (https://www.ncbi.nlm.nih.gov/geo/query/acc.cgi?acc=GSE301079) for scRNA-seq.

Research involving human participants, their data, or biological material

Policy information about studies with human participants or human data. See also policy information about sex, gender (identity/presentation), and sexual orientation and race, ethnicity and racism.

Reporting on sex and gender	N/A
Reporting on race, ethnicity, or other socially relevant groupings	N/A
Population characteristics	N/A
Recruitment	N/A
Ethics oversight	N/A

Note that full information on the approval of the study protocol must also be provided in the manuscript.

Field-specific reporting

Please select the one below that is the best fit for your research. If you are not sure, read the appropriate sections before making your selection.

☒ Life sciences ☐ Behavioural & social sciences ☐ Ecological, evolutionary & environmental sciences

For a reference copy of the document with all sections, see nature.com/documents/nr-reporting-summary-flat.pdf

Life sciences study design

All studies must disclose on these points even when the disclosure is negative.

Sample size	Sample sizes were determined using G*Power 3.1 analysis, validated as appropriate after data collection to yield actual effect sizes. When effect size was not predictable, an alternative method of using Resource equation based on value E was used and can be later validated by power analysis. These methods were followed as per Charan and Kantharia, 2013.
Data exclusions	No data were excluded unless there was a clear technical error, such as sample quality issues compromising RNA integrity and sequencing, tissue fixation issues and incomplete imaging of area of interest, which were rare events.
Replication	All experiments were at least replicated (performed independently) twice; most have been repeated more than three times.
Randomization	Samples were randomized, or if groups of samples were known a priori then groups were imaged in intercalated order.
Blinding	All genetic analyses were blinded until genotyped. All experiments that could be blinded were performed without a priori knowledge of type. Samples were blinded including during IHC staining and imaging, where controls and mutants were mixed and imaged/analyzed in random order; genotyping was performed only at the end to assign groups for comparison. For live imaging, homozygous mutants, which constitute only ~25% of the population, were pre-sorted to enrich their representation and also confirmed after data collection by genotyping. Imaging was performed in intermixed order between controls and mutants, followed by analysis, and genotyping was repeated at the end to verify all genotypes. Thus, all data were collected, and analyzed when possible, before genotyping, which was carried out using a PCR-based molecular assay.

Reporting for specific materials, systems and methods

We require information from authors about some types of materials, experimental systems and methods used in many studies. Here, indicate whether each material, system or method listed is relevant to your study. If you are not sure if a list item applies to your research, read the appropriate section before selecting a response.

Materials & experimental systems

n/a	Involved in the study
	☒ Antibodies
☒	Eukaryotic cell lines
☒	Palaeontology and archaeology
	☒ Animals and other organisms
☒	Clinical data
☒	Dual use research of concern
☒	Plants

Methods

n/a	Involved in the study
☒	ChIP-seq
	☒ Flow cytometry
☒	MRI-based neuroimaging

Antibodies

Antibodies used	The following primary antibodies were used in blocking solution: rabbit anti-smooth muscle actin (GTX100034, GeneTex) at 1:500, chicken anti-GFP (ab13970, Abcam) at 1:500, mouse anti-Pax7 (AB_528428, DSHB) at 1:100, and rabbit anti-CTSK (cathepsin K) at 1:500 (E7U5N, Cell Signaling Technology) followed by incubation with the appropriate secondary antibodies (AlexaFluor 647 goat anti-rabbit (Thermofisher A-21245), AlexaFluor 488 goat anti-chicken (Abcam ab150169), and AlexaFluor 647 goat anti-mouse (Thermofisher PIA28181) used at 1:500 to 1:2000. These are also detailed in Resources Table as Supplementary Data.
Validation	Use of antibodies were validated based on pattern of staining in control normal samples that matched description of reagent provided by the manufacturer and previously published data.

Animals and other research organisms

Policy information about studies involving animals; ARRIVE guidelines recommended for reporting animal research, and Sex and Gender in Research

Laboratory animals	Embryos from wild-type, mutant, and transgenic backgrounds were derived from: nlrc3st73, irf8 st95, myd88b1358, irg1/acod1 (this study), asc bcz82/nc303cs (this study), mrc1b sa18640 (this study), irg1-KI:GFP (knock-in, this study), irg1:GFP (tol2-based, this study), tnfa:GFPpd1028, "macro-rescue" mpeg1:nlrc3l24, lyz:mCherry, mpeg1:GFP, and mpeg1:BFP and raised at 28.5°C to stage for analysis. Genotyping primers and assays are described in Resources Table as Supplementary Data.
Wild animals	None, N/A.
Reporting on sex	N/A Only zebrafish embryos were used in this study for data collection, and sex is not determined until juvenile adult stages in zebrafish, therefore sex was not a variable in our study.
Field-collected samples	None, N/A.
Ethics oversight	This study was carried out in accordance with the approval of UNC-Chapel Hill Institutional Animal Care and Use Committee (protocols 19-132 and 22-103).

Note that full information on the approval of the study protocol must also be provided in the manuscript.

Plants

Seed stocks	n/a
Novel plant genotypes	n/a
Authentication	n/a

Flow Cytometry

Plots

Confirm that:

- [x] The axis labels state the marker and fluorochrome used (e.g. CD4-FITC).
- [x] The axis scales are clearly visible. Include numbers along axes only for bottom left plot of group (a 'group' is an analysis of identical markers).
- [x] All plots are contour plots with outliers or pseudocolor plots.
- [x] A numerical value for number of cells or percentage (with statistics) is provided.

Methodology

Sample preparation	Description is provided in the Materials and Methods.
Instrument	BD FACSMelody Cell Sorter
Software	BD FACSChorus software
Cell population abundance	99% purity was used based on the collection setting, population abundance is shown in Supplementary Figure 16, where immune cells generally represented less than 0.5% of the total events.
Gating strategy	Gating strategy is described with specific examples in Supplementary Figure 16.

- [x] Tick this box to confirm that a figure exemplifying the gating strategy is provided in the Supplementary Information.
